# Chromatin accessibility in canine stromal cells and its implications for canine somatic cell reprogramming

**DOI:** 10.1002/sctm.20-0278

**Published:** 2020-11-16

**Authors:** Maria Questa, Maryam Moshref, Robert J. Jimenez, Veronica Lopez‐Cervantes, Charles K. Crawford, Matthew L. Settles, Pablo J. Ross, Amir Kol

**Affiliations:** ^1^ Department of Pathology, Microbiology and Immunology School of Veterinary Medicine, University of California Davis Davis California USA; ^2^ Bioinformatics Core Facility University of California Davis Davis California USA; ^3^ Department of Animal Science University of California Davis Davis California USA

**Keywords:** ATAC‐Seq, cellular reprogramming, chromatin, dogs, induced pluripotent stem cells (iPSC), regenerative medicine

## Abstract

Naturally occurring disease in pet dogs is an untapped and unique resource for stem cell‐based regenerative medicine translational research, given the many similarities and complexity such disease shares with their human counterparts. Canine‐specific regulators of somatic cell reprogramming and pluripotency maintenance are poorly understood. While retroviral delivery of the four Yamanaka factors successfully reprogrammed canine embryonic fibroblasts, adult stromal cells remained resistant to reprogramming in spite of effective viral transduction and transgene expression. We hypothesized that adult stromal cells fail to reprogram due to an epigenetic barrier. Here, we performed assay for transposase‐accessible chromatin using sequencing (ATAC‐seq) on canine stromal and pluripotent stem cells, analyzing 51 samples in total, and establishing the global landscape of chromatin accessibility before and after reprogramming to induced pluripotent stem cells (iPSC). We also studied adult stromal cells that do not yield iPSC colonies to identify potential reprogramming barriers. ATAC‐seq analysis identified distinct cell type clustering patterns and chromatin remodeling during embryonic fibroblast reprogramming. Compared with embryonic fibroblasts, adult stromal cells had a chromatin accessibility landscape that reflects phenotypic differentiation and somatic cell‐fate stability. We ultimately identified 76 candidate genes and several transcription factor binding motifs that may be impeding somatic cell reprogramming to iPSC, and could be targeted for inhibition or activation, in order to improve the process in canines. These results provide a vast resource for better understanding of pluripotency regulators in dogs and provide an unbiased rationale for novel canine‐specific reprogramming approaches.

## Graphical abstract

Canine embryonic fibroblasts are successfully reprogrammed, but adult stromal cells remain resistant, likely due to epigenetic barriers. We performed assay for transposase‐accessible chromatin using sequencing on canine stromal cells and pluripotent stem cells, and identified distinct chromatin remodeling patterns. We identify candidate genes and transcription factor binding motifs that may be impeding adult cell reprogramming, and could be targeted to improve protocols in canines.

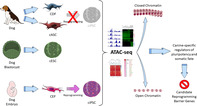

**Significance statement**

Poor understanding of canine‐specific regulators of cellular reprogramming and pluripotency maintenance impedes the use of naturally occurring disease in companion dogs for translational regenerative medicine research. In this work, the authors identify differences in global chromatin landscape that occur during cellular reprogramming and further define candidate barriers that prevent adult canine cells from reprogramming. Specifically, the genes and loci reported can be the basis of novel canine‐specific reprogramming protocols in future research efforts. The authors believe that these findings will be of great interest to the stem cell research community, especially to those seeking novel and realistic animal models for translational research.

## 1 INTRODUCTION

Induced pluripotent stem cells (iPSC) are pluripotent stem cells derived from somatic cells that have adopted an embryonic stem cell‐like phenotype. Somatic cells of murine, human, and other species can be reprogrammed by forced expression of pluripotency transcription factors (TF).^1^, ^2^ Cellular reprogramming technology has revolutionized the field of stem cell biology and regenerative medicine research, both by shifting our perspective of cellular development and differentiation, and by providing unprecedented opportunities for the creation of human disease models in a dish,^3^, ^4^ in vitro pharmacological, functional, and toxicity studies in laboratory‐derived human tissues,^5^ and regenerative medicine applications.^6^, ^7^

Time is ripe for the application of iPSC technology in regenerative medicine, although numerous translational challenges still need to be addressed before such innovative therapies can reach patients at a larger scale. The unique challenges associated with the translation of iPSC‐based cellular products to the clinic, along with the historically low approval rates of new candidate drugs that go into human clinical trials, call for a paradigm shift in translational biomedical research. Such a paradigm should be specifically designed to be able to test critical aspects such as safety,^8^ product scale‐up and delivery,^9^ and long‐term engraftment and immune compatibility,^10^ which are poorly modeled by traditional animal models.

Naturally occurring diseases in companion dogs are an extremely valuable and readily available resource as a preclinical model. Similarly to humans, dogs suffer from various complex multifactorial diseases, such as cancer, diabetes mellitus, cardiomyopathies, age‐related cognitive dysfunction, and neural damage, that are target for the development of iPSC‐derived cellular therapies.^11^ Dog population genetics mirror human population genetics with great variation across breeds and increasing homozygosity within breeds.^12^ The extended longevity of dogs is useful to test longer‐term immune compatibility and safety of stem cell derived cellular products.^13^ Furthermore, pet dogs in modern societies often receive excellent health care, hence allowing the use of veterinary medicine as a platform to conduct clinical trials, which mirror human clinical trials.

To date, six groups have reported the derivation of canine embryonic stem cells (cESC) from blastocyst‐stage embryos,^14^, ^15^, ^16^, ^17^, ^18^, ^19^ and nine groups have reported reprogramming of canine somatic cells into canine iPSC (ciPSC).^20^, ^21^, ^22^, ^23^, ^24^, ^25^, ^26^, ^27^, ^28^ However, candidate pluripotent cells often did not form teratomas upon injection into immune‐deficient mice, and no germline transmission has been demonstrated yet. Overall, lack of understanding of canine‐specific pluripotency maintenance regulators and reprogramming mechanisms thwarts robust and reproducible methods for canine somatic cell reprogramming.

We have successfully used previously described canine reprogramming protocols to reprogram canine embryonic fibroblasts (CEF) into stable ciPSC lines.^21^, ^29^, ^30^ On the other hand, adult cell types such as canine dermal fibroblasts (CDF) or canine adipose‐derived mesenchymal stem cells (cASC) remained resistant to reprogramming, despite effective transgene delivery, and multiple attempts, donors, and protocol modifications. Dynamic global chromatin remodeling underlies the reprogramming of somatic cells to iPSC and restructures chromatin accessibility across the entire genome. Such chromatin remodeling enables the inactivation of somatic loci and activation of pluripotency ones.^31^ We hypothesize that resistance to reprogramming in canine adult cells is due to a failure to close somatic‐fate loci, a failure to open pluripotency loci, or both, during the reprogramming process.

To test our hypothesis, we have determined global chromatin accessibility by assay for transposase‐accessible chromatin using sequencing (ATAC‐seq)^32^, ^33^ in two adult cell types (ie, CDF and cASC), CEF, CEF‐syngeneic ciPSC, and in cESC. This recently developed method maps open chromatin to genomic DNA regions, assisting in the determination of open loci and in the prediction of TF binding.^34^ We have identified genomic loci that “open” or “close” during the reprogramming of CEF into ciPSC, and further identified loci that are differentially accessible between the different cell types. Finally, we have identified 76 genes as potential canine‐specific somatic cell reprogramming barriers.

## 2 MATERIALS AND METHODS

Additional materials and methods can be found in Data S1.

### 2.1 Cell lines and culture

All animals and protocols in this study were approved by the Institutional Animal Care and Use Committee at the University of California (UCD) Davis, and all experiments conform to the relevant regulatory standards. CEF were derived from elective spays of pregnant uteri obtained from the Community Surgery Service at the UCD, School of Veterinary Medicine (SVM). Fetal age was determined prior to the spay by ultrasonographic evaluation by the attending clinician, or posteriorly by pulmonary maturation evaluation in hematoxylin and eosin‐stained slides, as previously described.^35^ After removal of the uterus and the ovaries, the uterine horns and the embryonic sacs were cut open with scalpels and the embryos were released by severing the umbilical cords. The head and viscera were removed, and the remaining stromal tissue was minced with scalpels and digested in 0.05% trypsin/EDTA (Gibco, Gaithersburg, MD, USA) at 37°C for 45 minutes. After washing the digested tissue with phosphate‐buffered saline, the pellet was plated and incubated in complete Dulbecco's modified Eagle's medium (DMEM), consisting of DMEM with 20% fetal bovine serum (Corning, Corning, NY, USA), 0.1 mM nonessential aminoacids, 2 mM GlutaMax, 1 mM sodium pyruvate, 100 U/mL penicillin, and 100 μg/mL streptomycin (Pen/Strep) (all Gibco).

CDF were derived from skin samples from deceased dogs obtained from the pathology service at the UCD SVM. All dog owners consented to unrestricted use of their dog's remains. With scalpel and scissors, fat and capillaries were scraped away from the dermis and skin was cut into small 2 to 4 mm^2^ sections and digested with collagenase type II (Worthington, Lakewood, NJ, USA) 1 mg/mL, at 37°C for 1 hour, with agitation. The digested cell suspension was centrifuged and the pellet filtered through a 100‐μm cell strainer; flowthrough was plated in complete DMEM. The remnant tissue sections were also plated in complete DMEM with the dermis side down.

cASC were a gift from Dr. Borjesson's laboratory and cultured as previously described.^36^

### 2.2 Generation of canine‐induced pluripotent stem cells

A number of 100 000 CEF were transduced with fresh OCT4‐KLF4‐SOX2‐IRES‐MYC (OKSIM)^29^ lentiviral media with 10 μg/mL polybrene (Millipore, Burlington, MA, USA). On day 2 posttransduction (PT), media was replaced with ciPSC media, and on day 4 PT cells were dissociated with TRypLE Express (Gibco) and plated in 10‐cm plates with fresh ϒ‐irradiated mouse embryonic fibroblasts (iMEF) at 100 to 200 000 CEF cells per plate. On days 5 to 10 PT, ciPSC colonies became visible and were picked between days 14 and 21 PT. The first two to three passages were performed manually; after three passages, clones were passaged by dissociation with collagenase type IV (Gibco), and plated onto fresh iMEF feeders, every 3 to 5 days. To induce differentiation, ciPSC and cESC colonies were dispersed with collagenase type IV, and then transferred to ultralow attachment plates (Corning) in DMEM. Cells were incubated in suspension for 7 days, during which they aggregated to form embryoid bodies (EB), which were then plated on 0.1% bovine gelatin (Sigma‐Aldrich, St. Louis, MO, USA)‐coated 24‐well plates and cultured for an additional 7 to 14 days.

### 2.3 Nucleofection of ciPSCs and luciferase assays

DNA constructs were electroporated into ciPSC with an Amaxa Nucleofector 2b Device (Lonza, Basel, Switzerland). Firefly and Renilla luciferase activity was assayed with Dual‐Glo Luciferase Assay System (Promega, Madison, WI, USA) following manufacturer's instructions. Bioluminescence was read in a Veritas Microplate Luminometer (Turner Biosystems, Sunnyvale, CA, USA), and ratio of Firefly over Renilla luciferase normalized to the same ratio of pGL3‐control wells.

### 2.4 Abbreviated ATAC‐seq chromatin accessibility assay and data analysis

See Data S1 for full ATAC‐seq methods.

Open chromatin DNA libraries were prepared from crude nuclei extracts by cell lysis, nuclei precipitation, and transposition for 60 minutes at 37°C. Libraries were then amplified with SsoFast EvaGreen Supermix (Bio‐Rad Laboratories, Hercules, CA, USA) and Nextera polymerase chain reaction (PCR) primers. To reduce guanine‐cytosine (GC) content and size bias, we monitored the PCR using quantitative Real Time PCR (qPCR) to stop amplification before saturation. Libraries were amplified for a total of 15 to 21 cycles, purified using Agencourt AMPure XP beads (Beckman Coulter, Brea, CA, USA), and sequenced in an Illumina HiSeq4000 system (Illumina, San Diego, CA, USA) in a paired‐end 150 bp run. At least 50 000 000 raw reads per cell type were obtained, except for cESC with only one cell line, which had approximately 40 000 000 raw reads. HTStream was used for data preprocessing, and fragments were mapped to the CanFam3.1 canine genome. Biological replicates were merged before peak calling. Differential openness analyses were conducted using the limma‐voom Bioconductor pipeline and peaks were annotated using the Bioconductor package ChIPseeker, version 1.20.0. Correlation plots, read‐depth heat maps, and profile plots were generated with deeptools. Hierarchical clustering was performed by distance calculation with Cluster 3.0 and visualized in Java TreeView. Gene ontology (GO) term classification was performed with PANTHER. TF motif enrichment analysis was performed with HOMER.

### 2.5 Statistical analysis

Pairwise Student's *t* tests and analysis of variance (ANOVA) tests were used to analyze statistical differences in all cases, except for the differential openness analysis (see Supplemental Information ‐ ATAC‐seq data analysis section). GraphPad Prism v8^37^ tools were used for both statistical analysis and graphical representation of results. A *P* value of <.05 was considered statistically significant.

## 3 RESULTS

### 3.1 Canine embryonic fibroblasts but not adult stromal cells can be reprogrammed to ciPSC

**FIGURE 1:**
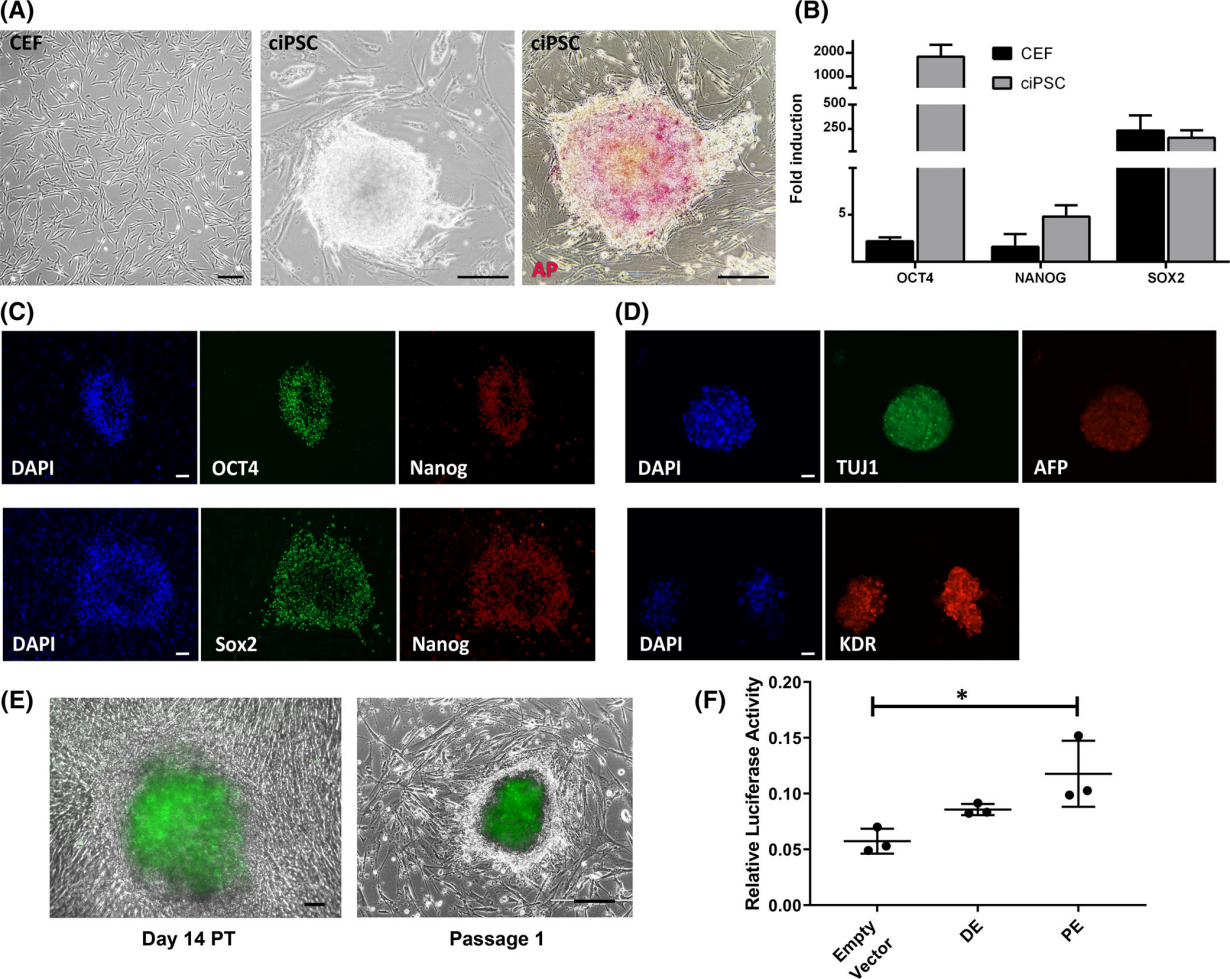
Generation of ciPSC and regulation of endogenous OCT4 expression in ciPSC. A, Morphology of CEF, syngeneic ciPSC, and AP activity in ciPSC. B, Quantitative reverse transcription PCR (RT‐qPCR) of CEF and ciPSC. All genes are normalized to canineperipheral blood mononuclear cells (PBMC). Bars are mean ± SEM. n = 15. C, Immunofluorescence of undifferentiated ciPSC, showing expression of OCT4, SOX2, and NANOG. D, Immunofluorescence of differentiated ciPSC‐derived embryoid bodies, showing expression of lineage markers TUJ1, KDR, and AFP. Scale bars are 200 μm. E, Representative images of *OCT4‐2A‐eGFP‐PGK‐Puro* edited and reprogrammed CEF, showing enhanced Green Fluorescent Protein (eGFP) expression (green) as a reporter for *OCT4*, at day 14 posttransduction, and on day 1 after passage 1. F, Relative luciferase expression (Fluc/Rluc) controlled by the human cytomegalovirus (CMV) minimal promoter and the *OCT4* distal enhancer (DE) or proximal enhancer (PE) on ciPSC.*P* < .05 for PE vs empty vector, as shown by one‐way ANOVA and Friedman multiple comparisons test. Scale bars are 200 μm. ANOVA, analysis of variance; AP, alkaline phosphatase; CEF, canine embryonic fibroblasts; ciPSC, canine‐induced pluripotent stem cells

We began our study with the transduction of the four Yamanaka factors (OCT4, SOX2, KLF4, and MYC; OSKM) into low passage CEF, cASC, and CDF. The characteristics of the dogs from which the cell lines were derived are detailed in Table S1. We show here that reprogrammed CEF formed colonies of ciPSC with stem cell‐like morphology and high alkaline phosphatase (AP) activity (Figure 1A). ciPSC colonies showed induced expression of core pluripotency genes *OCT4*, *NANOG*, and *SOX2* (Figure 1B,C) and, when spontaneously differentiated via EB formation, generated cells of the three germ layers, as shown by ectoderm, mesoderm, and endoderm lineage marker expression (Figures 1D and S1). ciPSC further silence transgene expression in later passages (Figure S2).

In order to establish the bona fide reprogramming of CEF and study the transcriptional regulation of endogenous OCT4 expression, we generated a genetically modified OCT4‐eGFP reporter system, tagging the endogenous *OCT4* locus with eGFP in CEF. Upon OSKM transduction of the modified CEF line, eGFP expression was observed starting on day 4 PT, indicating endogenous *OCT4* expression, and was consistently visualized in formed ciPSC colonies on p0 and in further passages (Figure 1E). *OCT4* transcription from its endogenous promoter is enhanced by either the distal enhancer (DE) in naïve stem cells or the proximal enhancer (PE) in primed stem cells.^38^, ^39^ To study the transcriptional regulation of the *OCT4* locus in ciPSC we cloned the canine DE and PE in a reporter plasmid upstream of a minimal promoter controlling the expression of luciferase (luc) (Figure S3). The construct was nucleofected into CEF‐derived ciPSC, and luc activity assays were conducted, indicating the OCT4 locus in ciPSC is controlled by the PE and not the DE, suggesting a primed state for these iPSC (Figure 1F).

cASC and CDF, as well as testicular and ovarian fibroblasts, did not yield stable colonies, even though the efficiency of infection was comparable to that of infected CEF (Figure S4).

### 3.2 ATAC‐seq analysis identifies distinct cell type clustering pattern

**FIGURE 2:**
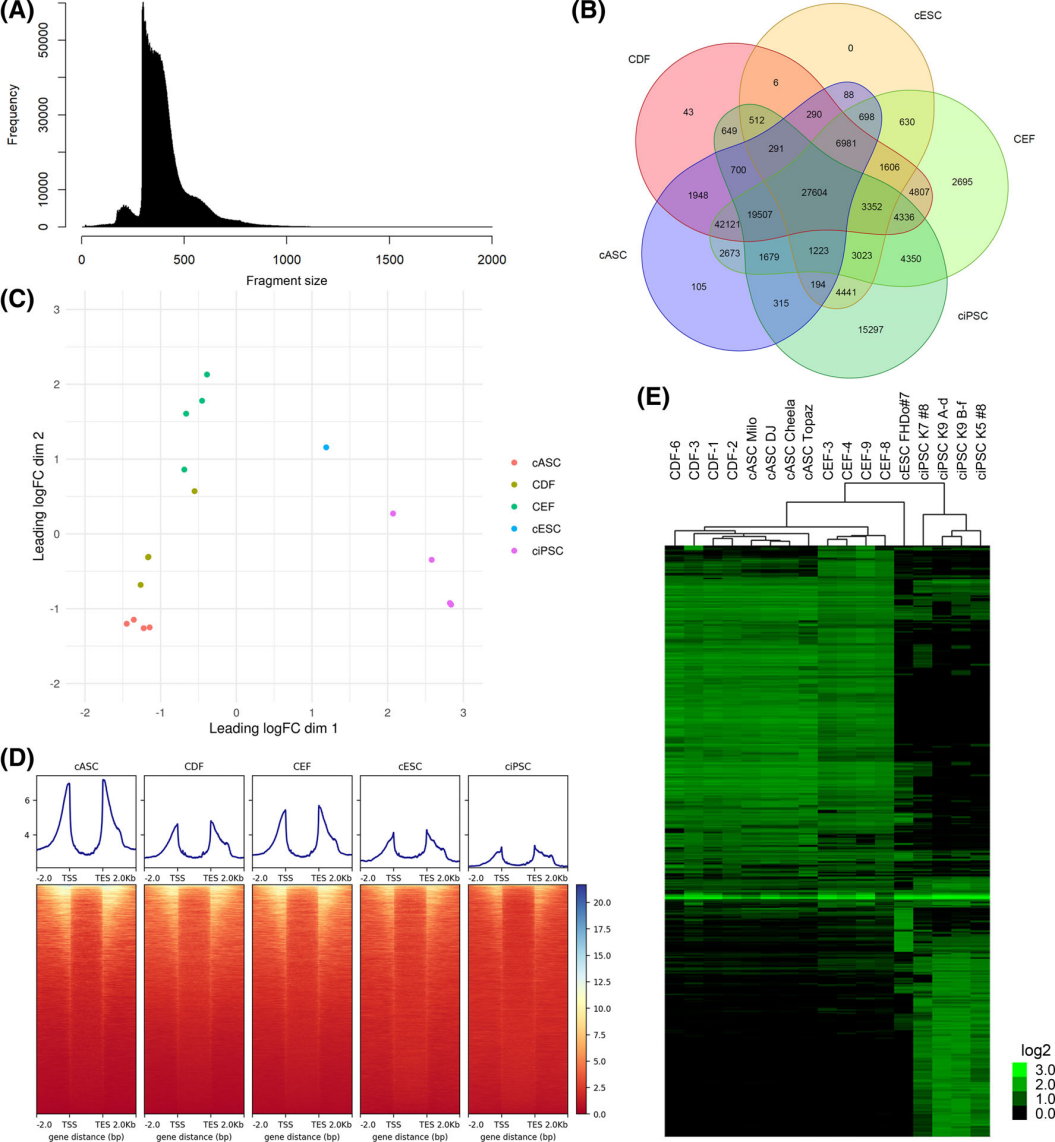
ATAC‐Seq shows chromatin accessibility differences between adult and embryonic stromal cells, and canine pluripotent cells. A, Representative histogram of the frequency distribution of DNA library fragment size from ATAC‐sequencing. B, Venn diagram of the distribution of ATAC‐seq peaks for the cell types studied. C, Two‐dimensional scaling plot of the relative distances for ATAC‐seq peaks between cell types CDF, cASC, CEF, ciPSC, and cESC. D, Read‐depth heat map of the whole data set of ATAC‐seq peaks. Representation from 2 kb upstream to 2 kb downstream of each loci. All genes represented from TSS to TES. E, Pearson correlation heat map of a representative sample of*all peaks*, downsized to enhance processing efficiency, for all the cell type data sets. Each column is a cell type and each row is an ATAC‐seq peak. Color scale shows relative ATAC‐seq peak signal, on a log_2_base. ATAC‐Seq, assay for transposase‐accessible chromatin using sequencing; cASC, canine adipose‐derived mesenchymal stem cells; CDF, canine dermal fibroblasts; CEF, canine embryonic fibroblasts; cESC, canine embryonic stem cells; ciPSC, canine‐induced pluripotent stem cells; TES, transcription end site; TSS, transcription start site

We hypothesized that resistance of canine adult stromal cells (ie, CDF and cASC) to OSKM‐mediated cellular reprogramming is due to a failure to close somatic‐fate loci, a failure to open pluripotency loci, or both, during the reprogramming process. To test our hypothesis, we studied the chromatin accessibility landscape of canine stromal and pluripotent cells by ATAC‐seq. Specifically, we studied two adult stromal cell types (ie, CDF and cASC), CEF, CEF‐syngeneic ciPSC, and cESC. We sequenced four different cell lines of each cell type mentioned, except for cESC of which we only had one cell line available. The following are results from the analysis of all the peaks in the complete data set. The frequency of sequenced fragment size followed the expected pattern for an ATAC‐seq library with periodical peaks corresponding to the nucleosome‐free regions (NFR, 100 bp and under) and mononucleosome, dinucleosome, and trinucleosome (200, 400, and 600 bp approximately, respectively),^33^ in this case with most of the fragments being 200‐400 bp, which is consistent with an enrichment in mononucleosome and dinucleosome (Figure 2A). A Venn diagram of the peaks shared by all cell types (Figure 2B) is a first approach at identifying global patterns of chromatin openness. It shows a markedly higher number (4350) of shared peaks between ciPSC and CEF, compared with those shared between ciPSC and CDF and/or cASC (649 and 315, respectively). Two‐dimensional clustering analysis further shows that the different cell types form distinct clusters that match their different biologic origin (Figures 2C). We further hypothesized that ATAC‐seq peaks would be overrepresented in regulatory areas around the transcription start site (TSS) and transcription end site (TES). Our analysis confirms that in all cell types, peaks were overrepresented in TSS and TES areas compared with exons and introns (Figure 2D). Finally, we used Pearson correlation unsupervised hierarchical clustering of all peaks obtained, to identify cell type clustering and distances, which indicate that the primary node of separation was between pluripotent stem cells and stromal cells, and a secondary node separated CEF from the adult stromal cells (ie, ASC and CDF) (Figure 2E).

When we studied the genomic element distribution of differential peaks we found that proper regulatory areas (promoter and 2 kb upstream and downstream), exons and introns, were enriched in peaks with the highest fold change (Figure S7), indicating that using this subdata set is more effectual for the current study of chromatin accessibility. This subset composed of peaks annotated as promoter, upstream 0 to 2 kb, downstream 0 to 2 kb, exons and introns was used in all the analyses that follow.

### 3.3 Chromatin remodeling during CEF reprogramming

**FIGURE 3:**
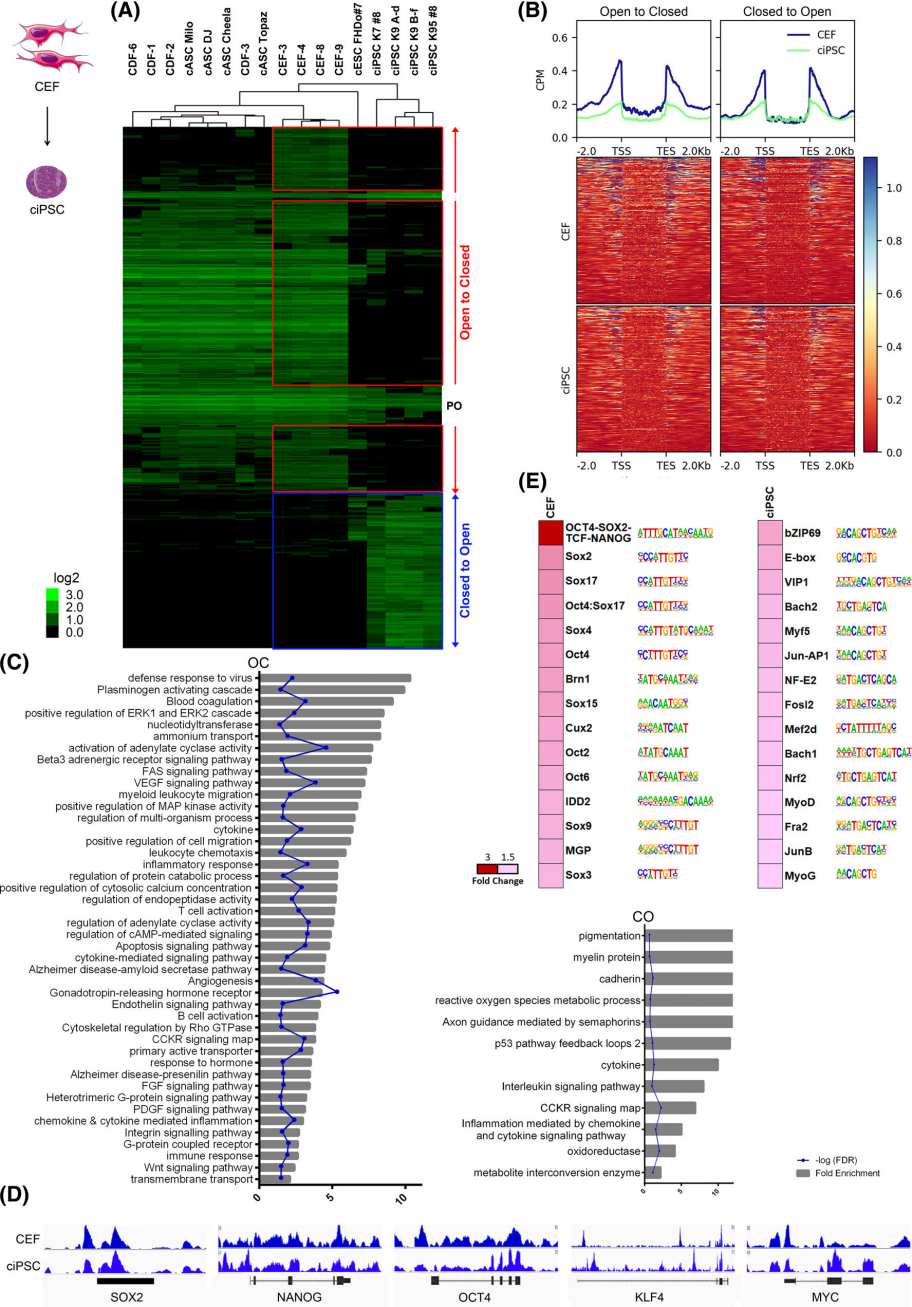
Genomic accessibility landscape remodeling during canine stromal cell reprogramming. A, Pearson correlation heat map of 2403 peaks with differential openness when comparing embryonic stromal cells (ie, CEF) vs ciPSC, representing the CEF‐ciPSC transition, for all cell type data sets. Each column is a cell type and each row is an ATAC‐seq peak. Color scale shows relative ATAC‐seq peak signal, on a log_2_base. B, Read‐depth heat map of OC and CO gene clusters, for the CEF *vs* ciPSC data set. Genes represented from TSS to TES. C, GO term enrichment by PANTHER overrepresentation test with fold enrichment >2, *P* < .05, and FDR <0.05. Showing fold enrichment and corresponding FDR, for the CO and OC gene groups. D, Selected Integrative Genome Viewer (IGV) genomic views of ATAC‐seq data for stemness genes*SOX2*, *NANOG*, *OCT4*, *KLF4*, and *MYC*. All genome view vertical scales were group autoscaled to normalize for read‐depth. Genes are oriented 5′ to 3′ and graphed from 2 kb upstream of the TSS to 2 kb downstream of the TES. E, Open TF motif discovery for pairwise comparison CDF‐cASC vs CEF‐ciPSC. TF families and motifs are indicated on the right of the fold‐change heat map. E, Open TF motif discovery for pairwise comparison CEF vs ciPSC. TF families and motifs are indicated on the right of the fold‐change heat map. ATAC‐Seq, assay for transposase‐accessible chromatin using sequencing; cASC, canine adipose‐derived mesenchymal stem cells; CDF, canine dermal fibroblasts; CEF, canine embryonic fibroblasts; ciPSC, canine‐induced pluripotent stem cells; CO, closed to open; FDR, false discovery rate; GO, gene ontology; OC, open to closed; TES, transcription end site; TSS, transcription start site

In order to define the global chromatin remodeling that occurs during CEF reprogramming into ciPSC, we compared ATAC‐seq results from CEF vs CEF‐syngeneic ciPSC, by unsupervised hierarchical clustering and heat map plotting (Figure 3A). Two clusters emerged from this analysis: the “Open to Closed” (OC) peaks cluster, and the “Closed to Open” (CO) peaks cluster. OC and CO peaks plotted across all cell types showed that adult stromal cells followed a similar pattern to CEF cells, except for a cluster of peaks that is closed in adult stromal cells while open in CEF (Figure 3A). As expected, the permanently open group contained primarily housekeeping genes such as *GAPDH* or *RPL4/14* (data not shown). The group of loci that were differentially open in ciPSC compared with CEF (CO) included well‐validated pluripotency associated genes such as *SMAD3* and *ALPL*, as well as candidate novel canine pluripotency‐associated genes such as *WNT5A*, *AVPR1*, or *BMP7*. Finally, genes that were differentially closed in ciPSC compared with CEF (OC) included many genes that are known to enhance the somatic fate, such as *SYNGR2* and *VEGFA* as well as candidate novel canine somatic fate genes such as *GTF2A1L*, *GSTA4*, or *MMP3*. In both CO and OC groups, different genomic areas suffered chromatin accessibility changes in both directions (open to closed and vice versa). First, the cluster peaks lists were converted to genes lists from annotation, in order to construct a read‐depth heat map along the gene loci from TSS to TES (Figure 3B). This shows the same enrichment of peaks flanking the TSS and TES as seen above for the whole data set, with OC genes more open in CEF than in pluripotent cells in all genomic areas, and CO genes more open in pluripotent cells around areas flanking both the TSS and TES, as compared with the same cell population for OC genes. In addition, CEF OC genes are more closed than CO genes, especially in the coding areas. Next, enrichment of GO terms for the OC and CO groups (Figure 3C) revealed that amongst the pathways that are enriched in the OC group are immunity‐related genes, and the Wnt, VEGF, PDGF, and FGF pathways, all related to cell identity establishment or maintenance, among many others. This is in alignment with expected chromatin remodeling during reprogramming, but the fact that we did not observe an opening or enrichment of classic stemness genes between CEF and ciPSC, is likely due to these being already open enough in CEF, hence chromatin accessibility does not need to be modified significantly for reprogramming (Figure 3D). This might not be the case when the donor cells are CDF or cASC, where stemness genes are not accessible, but it was impossible to assess this at the ciPSC level, since CDF and cASC did not form established ciPSC lines that we could extract material from to study. Genes enriched in the CO group include oxidoreductase and reactive oxygen species metabolism‐related genes, which underlines the importance of oxygen metabolism in stem cells, as well as cadherin, the p53 pathway and cytokines. Finally, we studied the TF motif patterns in CEF before and after reprogramming to ciPSC (Figure 3E) and found that TF motifs for stemness factors such as OCT4, SOX2, and NANOG, as well as other *OCT* and *SOX* genes are already open in CEF, and differentially more open than in ciPSC, making them receptive to the introduction of OSKM. Surprisingly, ciPSC seem to have an enrichment for open TF motifs more commonly related to cell identity maintenance like Jun‐AP1, Mef2D, MyoD, or E‐box, when compared with CEF.

### 3.4 Chromatin landscape differences between adult and embryonic stromal cells

**FIGURE 4:**
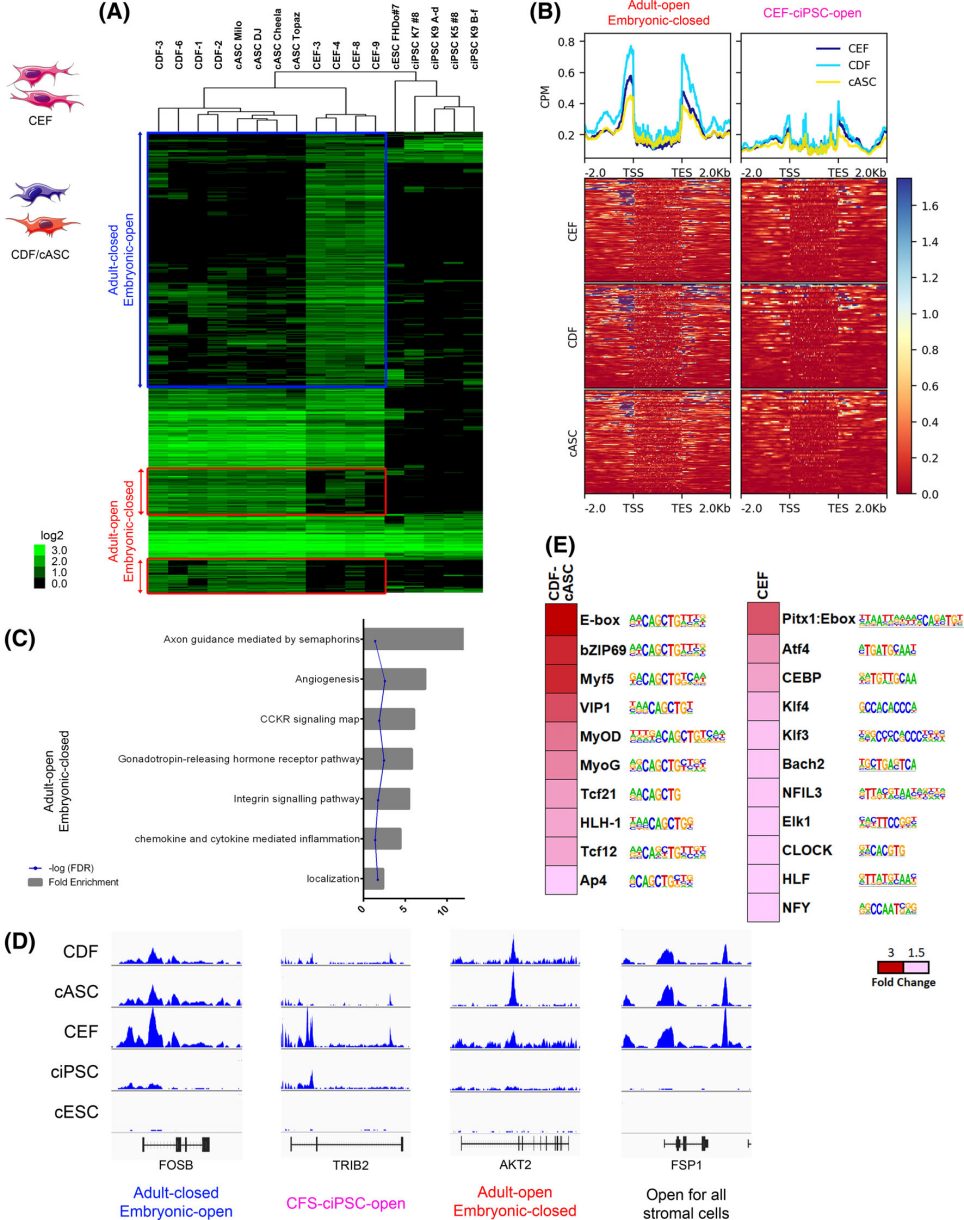
Genomic accessibility landscape in canine adult and embryonic stromal cells. A, Pearson correlation heat map of 976 peaks with differential openness when comparing embryonic stromal cells (ie,: CEF) vs adult stromal cells, for all the cell type data sets. Each column is a cell type and each row is an ATAC‐seq peak. Color scale shows relative ATAC‐seq peak signal, on a log_2_base. B, Read‐depth heat map of clusters III and IV, for the CDF‐cASC vs CEF data set. C, GO term enrichment by PANTHER overrepresentation test with fold enrichment >2,*P* < .05, and FDR <0.05. Showing fold enrichment and corresponding FDR, for the “Adult‐open Embryonic‐closed” group. No enrichment found for “CEF‐ciPSC‐open” group. D, Selected Integrative Genome Viewer (IGV) genomic views of ATAC‐seq data for representative genes from each cluster. All genome view vertical scales were autoscaled to normalize for read‐depth. Genes are oriented 5′ to 3′ and graphed from 2 kb upstream of the TSS to 2 kb downstream of the TES. E, Open TF motif discovery for pairwise comparison CDF‐cASC vs CEF. TF families and motifs are indicated on the right of the fold‐change heat map. ATAC‐Seq, assay for transposase‐accessible chromatin using sequencing; cASC, canine adipose‐derived mesenchymal stem cells; CDF, canine dermal fibroblasts; CEF, canine embryonic fibroblasts; ciPSC, canine‐induced pluripotent stem cells; FDR, false discovery rate; GO, gene ontology; TES, transcription end site; TF, transcription factors; TSS, transcription start site

We analyzed ATAC‐seq peaks that were significantly more open or closed when comparing CDF‐cASC to CEF, by unsupervised hierarchical clustering (Figure 4A). Genomic patterns derived from this analysis were grouped into clusters, which when overlaid with all the cell lines studied, resulted in similar hierarchical organization to the one observed before (Figures 2C‐E). Furthermore, the read‐depth heat map for the genes derived from these peaks cluster groups (Figure 4B) shows the same peak enrichment flanking the TSS and TES, as previously observed. In addition, we have identified genes that are more open in adult stromal cells as opposed to CEF, especially in gene‐flanking areas, as observed in Figure 4B, top panel graphs. This group was labeled “Adult open/Embryonic closed”. This can also be seen in Figure 4A where these peaks appear in green for CDF and cASC, and mostly black for CEF. Meanwhile, CEF present an intermediate chromatin openness level in regulatory areas, which is between that of CEF and cASC, but are clearly more closed in coding areas (Figure 4B, top panel graphs). Moreover, chromatin accessibility in pluripotency core genes was diminished in CDF, when compared with CEF, especially in *SOX2*, *NANOG*, and *OCT4* (Figure S6), proving that CEF indeed might be primed for pluripotency, at least more so than CDF. GO term analysis of this CEF vs CDF‐cASC comparison (Figure 4C) showed a larger proportion of open genes in adult stromal cells related to axon guidance, angiogenesis, GnRH release, and inflammation, all related to an ultimately differentiated state, as well as genes related to general functions such as signaling. Among these were *PRKDC*, *DMD*, *RHOA*, *BAX*, *MMP11*, *ANGPT1*, *JAK1*, *VEGFA*, *VASP*, *STAT6*, *AP3S2*, *ANGPT1*, and *BMP4*. There was no enrichment of GO terms in “CEF‐ciPSC‐open”‐associated genes. Representative ATAC‐seq profiles for the defined clusters can be seen in Figure 4D. We also studied the enrichment of TF motifs in the CDF‐cASC group vs CEF (Figure 4E), and found TF motifs for well‐known differentiation‐associated TF, like E‐box, MyoD/G, Tcf21/12 to be enriched in the adult subset, while the CEF subset was enriched for both differentiation‐related motifs like Pitx1:E‐box, as well as embryonic‐associated motifs like Klf3/4, CEBP, and NFY.

### 3.5 Differential chromatin accessibility analysis identifies candidate “fail‐to‐close” and “fail‐to‐open” loci in adult stromal cells

**FIGURE 5:**
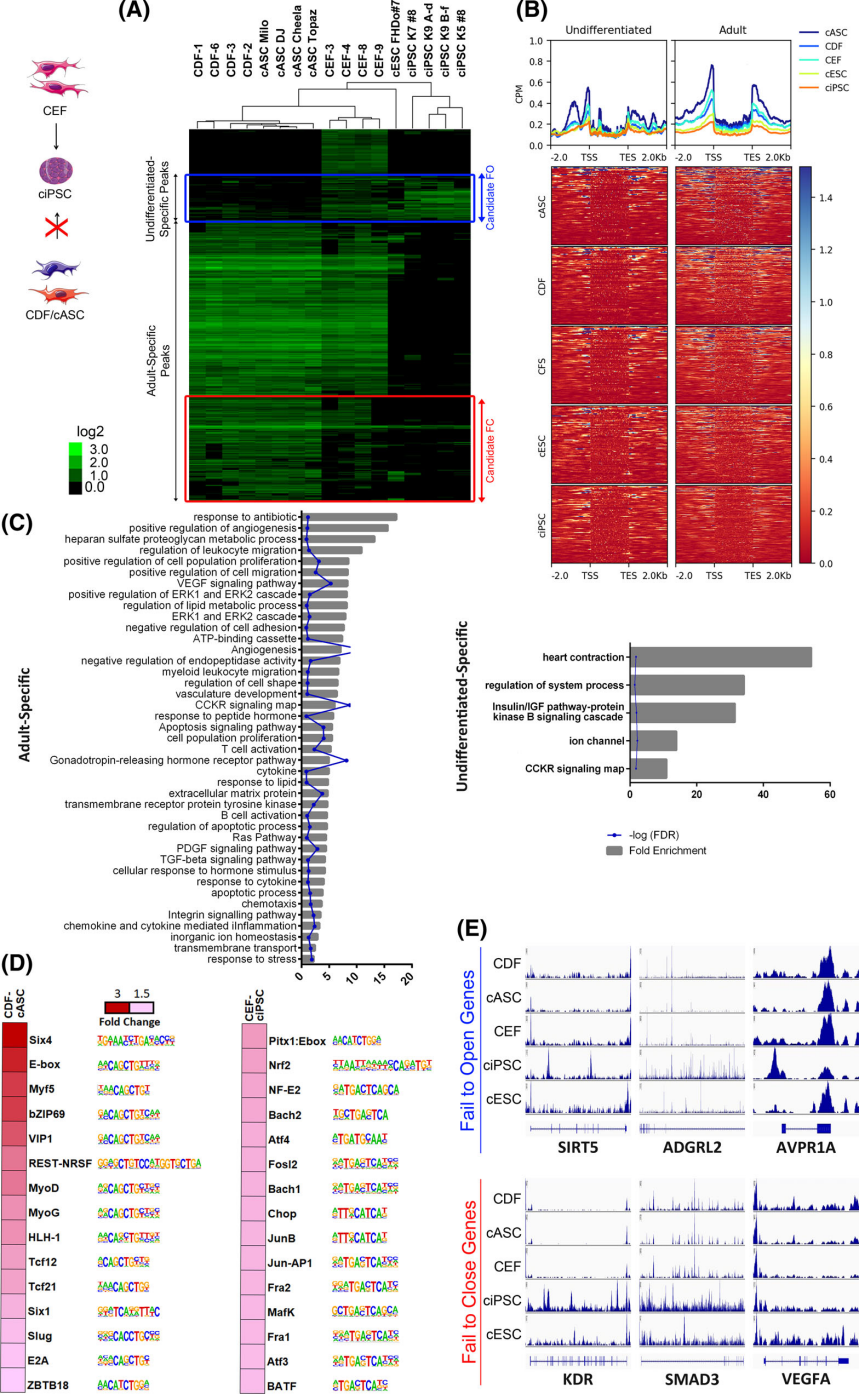
Candidate genetic barriers to the reprogramming of adult canine stromal cells. A, Pearson correlation heat map of 935 peaks with differential openness when comparing adult stromal cells vs embryonic stromal cells (CEF) and ciPSC, representing the reprogramming barriers for generation of ciPSC from CDF and cASC but not from CEF, for all the cell type data sets. Each column is a cell type and each row is an ATAC‐seq peak. Color scale shows relative ATAC‐seq peak signal, on a log_2_base. B, Read‐depth heat map of adult and undifferentiated‐specific gene clusters, for the CDF‐cASC vs CEF‐ciPSC data set. C, GO term enrichment by PANTHER Overrepresentation test with Fold Enrichment >2,*P* < .05, and FDR <0.05. Showing fold enrichment and corresponding FDR, for the adult and undifferentiated‐specific gene clusters. D, Open TF motif discovery for pairwise comparison CDF‐cASC vs CEF‐ciPSC. TF families and motifs are indicated on the right of the fold‐change heat map. E, Selected IGV genomic views of ATAC‐seq data for representative genes from candidate FC and candidate FO gene lists. All genome view vertical scales were autoscaled to normalize for read‐depth. Genes are oriented 5′ to 3′ and graphed from 2 kb upstream of the TSS to 2 kb downstream of the TES. ATAC‐Seq, assay for transposase‐accessible chromatin using sequencing; cASC, canine adipose‐derived mesenchymal stem cells; CDF, canine dermal fibroblasts; CEF, canine embryonic fibroblasts; ciPSC, canine‐induced pluripotent stem cells; FC, fail to close, FO, fail to open; FDR, false discovery rate; GO, gene ontology; TES, transcription end site; TF, transcription factors; TSS, transcription start site

In order to study which genes might represent barriers to reprogramming, we analyzed comparison of peaks from adult stromal cells vs CEF and ciPSC. We defined the new “Adult‐Specific” and “Undifferentiated‐Specific” clusters, as the peaks that were significantly more open in adult stromal cells as opposed to CEF and ciPSC, and the peaks that were significantly more open in CEF and ciPSC as opposed to adult stromal cells, respectively (Figure 5A). Peaks that were closed in adult cells but open in CEF and ciPSC were considered “fail‐to‐open” (FO) peaks, and peaks that were open in adult cells but close in CEF and ciPSC were considered “fail‐to‐close” (FC) peaks. The read‐depth heat map for the genes derived from the “Adult” and “Undifferentiated‐Specific” peak groups (Figure 5B) shows the same enrichment of peaks flanking the TSS and TES we have seen before, especially for Adult peaks, while the Undifferentiated peaks show a more scattered pattern of chromatin accessibility. Adult‐associated genes were more open in stromal cells than in pluripotent cells, and especially more open in CDF; undifferentiated‐associated genes show a change in chromatin accessibility pattern in this case, displaying a more concentrated openness in regulatory areas before the TSS and after the TES, as compared with their condition for adult genes. GO term enrichment analysis (Figure 5C) showed the most significantly overrepresented GO terms in the “Adult‐Specific” gene cluster were related primarily to cell identity establishment and maintenance, gene regulation processes such as nucleic acid binding, and pathways such as VEGF, PDGF, and EGF, underlining how a failure to close loci related to cell commitment can have be detrimental to reprogramming. TF motif analysis (Figure 5D) revealed that several differentiation‐associated TF motifs, like E‐box, MyoD/G, NF‐E2, or Tcf21/12 were enriched in CDF‐cASC, as seen in other comparisons including this group. While, on the other hand, CEF‐ciPSC were enriched in TF motifs related to stem cell maintenance like Nrf2, Bach1, and Atf3, but also some cell identity motifs like AP1, Fosl, and Jun.

Finally, with the aim of obtaining a restricted list of possible reprogramming barrier genes, we cleaned up the FC and FO gene clusters defined before, by overlapping them with the OC or CO gene groups, respectively, obtained from the analysis shown in Figure 4, and by filtering the resulting genes by GO term. We defined the “Candidate FC” group, derived from the filtered FC group, as the cluster of peaks that is open in adult stromal cells, and less open or closed in CEF, ciPSC, and cESC; and the “Candidate FO” group, derived from the filtered FO group, as the cluster of peaks that is closed in adult stromal cells, but open in CEF, ciPSC, and cESC. The proposed Candidate FC and FO gene barriers are shown in Table 1, and representative ATAC‐seq profiles in Figure 5E. Barrier gene candidates include lineage associated genes that may fail to close, such as *ALCAM*, *VEGFA*, *KDR*, and genes that may fail to open such as *NOX3*, *SIRT5*, or *BCL2*.

**TABLE 1:** Candidate reprogramming barrier genes

Candidate FC			Candidate FO
ADGRE1	GUSB	PRKDC	ACAN
ALCAM	HIPK2	PSMB7	ADGRL2
ANGPT1	HMGA1	QKI	AVPR1A
AOX2	HRAS	RAB10	B3GAT2
BCAN	IL2RA	RAB22A	BCL2
BCL2L1	JUP	RBM12	CTSC
BMPR1B	KCNMB1	RECK	DCT
CAV1	KDR	RETREG1	DSG3
CCL17	LEPR	SERPINE1	GMPS
COMMD1	MAPK1	SERPINH1	GNAQ
CPT1A	MAPT	SLC6A2	LIPC
CRYBA1	MMP11	SMAD3	NOX3
DAG1	NDRG1	SREBF1	PNPLA1
DDX39B	NFKB1	STK38L	PSEN1
DKK3	NUDT3	SULF1	SIRT5
DPT	PDX1	UBE2N	SLC7A1
EIF3L	PLCE1	UNK	SPINK5
FBN1	PPARG	VEGFA	TACR1
GHR	PPP3CA	XYLT1	TACR3

Abbreviations: FC, fail to close; FO, fail to open.

## 4 DISCUSSION

While canine somatic cell reprogramming via forced expression of pluripotency TF has been previously described, reported cells often lacked critical qualities of iPSC and reports have been sporadic and inconsistent, reflecting lack of a deeper, specific‐understanding of reprogramming mechanisms, and pluripotency regulators. Canine somatic cell nuclear transfer (SCNT) as a means for cell reprogramming is a more mature and robust technology with numerous live puppies born via such an approach.^40^, ^41^ Nonetheless, no ESC lines have been generated by SCNT in dogs.

Here, we demonstrate the ability to reprogram CEF to ciPSC, and report the inability of canine adult stromal cells to generate stable ciPSC colonies. Cellular age could have an effect on reprogramming efficiency, but this was not the case in the current study. CEF had a tendency to proliferate faster, and while higher proliferative rate is associated with increased reprogramming efficiency, which could explain the tendency of CEF to be reprogrammed more efficiently, there were no statistical differences in PDT among the cell types studied (Figure S5). Furthermore, as is the case with murine strains,^42^ there are no literature reports that suggest there is a canine breed dependence for somatic cell reprogramming, and although we did not observe any influence of breed in reprogramming, this cannot be ruled out.

Through the generation of an OCT4‐eGFP reporter system, we show that the endogenous OCT4 locus is engaged during reprogramming of CEF, and that this is controlled mainly from the PE enhancer,^43^, ^44^ which suggests that our reprogrammed ciPSC are in a primed pluripotency state. Furthermore, to study the difference in reprogramming capabilities between embryonic and adult canine stromal cells, we performed effective and reproducible ATAC‐seq sequencing and data analysis on different types of canine cells. Our data analysis approach allowed us to identify groups of genes that may be involved in the inability to reprogram adult stromal cells.

We initially studied the process of reprogramming of CEF to ciPSC, identifying major pathways that change during this process. Among them, the Wnt, VEGF, FGF, and PDGF pathways were of interest.^45^, ^46^, ^47^ Notable loci expected to close that indeed did were *AFP*, *ANGPT1*, *AR*, and *KDR*, all markers of committed lineages.^48^, ^49^, ^50^, ^51^ We noted that OC changes were the most represented in our analysis, underlining the importance of appropriate somatic loci shutdown for reprogramming. When we compared ATAC‐seq data between embryonic fibroblasts and adult stromal cells we found that some loci were open in CEF as opposed to CDF‐cASC, such as FGFR2 and *IGF1*, both of which have roles in embryonic development and stem cell self‐renewal.^52^, ^53^ These could be targets that need to be opened or stimulated during reprogramming of CDF‐cASC.

Certain genes that we have identified as candidate FO genes in the current study, such as *SIRT5*, are strongly related to DNA repair, intracellular homeostasis, redox homeostasis, DNA transcription regulation and microenvironment regulation, all key functions during the process of genetic reprograming.^54^, ^55^, ^56^, ^57^, ^58^, ^59^ Such FO genes could be specifically targeted for activation by techniques such as the CRISPRa system,^60^ or with small molecule activators of specific pathways.

Our data further identified the genes *ALCAM*, *BMPR1B*, and *DAG1* as candidate FC genes. While there is limited reports of *ALCAM* and no reports of dystroglycans' like *DAG1* effects on reprogramming in the literature,^61^, ^62^ several other adhesion molecules have been shown to have positive as well as detrimental effects in reprogramming of murine cells.^63^, ^64^, ^65^ *ALCAM* and *DAG1* could be novel reprogramming barriers in the canine model, as well as the human and murine models. The *BMP* receptors or their associated pathways were previously reported to be involved in somatic cell reprogramming^66^, ^67^, ^68^ and could be barriers to canine specific cell reprogramming.

Interestingly, some TF motifs that were enriched in CEF and ciPSC as opposed to adult stromal cells were FOSL2, Jun‐AP1, and JUNB, which have been shown to be involved in identity maintenance, proliferation, immune response, and cell death.^69^, ^70^ Interestingly, several members of the AP‐1 family have previously been shown to inhibit reprogramming,^71^ and together with the fact that data showed no increased chromatin accessibility for these loci, we hypothesize that the TF motif enrichment is not related to increased AP‐1 formation and/or activity. These motifs might confer different TF functions in canines, as opposed to the human/murine models. In conclusion, these genes constitute possible reprogramming barriers, whose expression should be investigated within this model, and can be targets in interference experiments, to the end of identifying a pathway that when blocked can serve as a strong reprogramming enhancer.

TF motif discovery for CEF vs ciPSC showed surprising results, with motifs for OCT4, SOX2, and NANOG enriched in CEF instead of ciPSC, as expected, OCT4 being one of the essential pioneering factors needed for cellular reprogramming in most models studied.^72^ When we performed TF motif discovery for adult stromal cells against embryonic fibroblasts we found, as expected, that TF family motifs enriched in adult stromal cell peaks are related to somatic loci activation and lineage identity development and maintenance, such as MyoD/G, involved in muscle gene expression and cell determination; and Tcf21, associated with AP‐1 binding.^73^ On the other hand, the motifs enriched in CEF included Klf4 and NFY motifs, both associated with pluripotency.^74^ TF motif discovery for CEF vs ciPSC showed surprising results, with motifs for OCT4, SOX2, and NANOG enriched in CEF instead of ciPSC, as expected. We hypothesized that the latter findings reflect the “reprogram primed” state of CEF, though additional functional validation is required. Lastly, when we studied the motifs enriched in adult stromal cells against the motifs shared by CEF and ciPSC, we found that motifs associated with pluripotency maintenance were enriched in sequences differentially open in ciPSC, such as Nrf2,^75^ Bach1^74^ and Atf3, a target of Myc, that could be related to pluripotency maintenance through cell proliferation effects. These motifs could be targeted for chromatin aperture, in order to improve a canine‐specific adult stromal cell reprogramming protocol.

Moreover, genome annotation in a non‐model species like the dog is still suboptimal. An astounding 84 Mb of canine transcribed sequence is not found in the existing Ensembl canine reference. In addition, one study reports finding most ATAC‐seq peaks in promoter areas,^76^ whereas we found most peaks in distal intergenic areas. The inaccuracy of the genome annotation contributes to difficulty in assignment of genomic areas to appropriate genes or genomic functions. In particular for the dog genome, annotation for a high amount of loci and regulatory regions is only predicted, not precise, or directly inexistent, generating not only the need for manual data analysis or curation, but also the inability to detect associations. Continued work on the species will generate more interest, more research, and more investment in resequencing the species' genome and performing more functional annotation. In addition, different genomic areas of a given gene suffered bidirectional chromatin accessibility changes; this speaks about a very complex chromatin rearrangement during reprogramming that is probably based on both positive and negative enhancer areas being differentially accessible.

## 5 CONCLUSIONS

Our data provide a deeper understanding of nuclear chromatin remodeling during cellular reprogramming in dog cells, and define candidate barriers for somatic cell reprogramming. Such candidate reprogramming barriers could be the target of future studies that aim to generate a better understanding of the regulatory networks that govern canine pluripotency. The uncovering of particular targets to facilitate cellular reprogramming to ciPSC will support the generation of robust ciPSC for translational research in a more efficient manner.

## CONFLICT OF INTEREST

The authors declared no potential conflicts of interest.

## AUTHOR CONTRIBUTIONS

M.Q.: conception and design, collection and assembly of data, data analysis and interpretation, manuscript writing, final approval of manuscript; M.M., R.J.J., V.L.‐C., C.K.C., M.L.S.: collection of data, final approval of manuscript; P.J.R.: design, data analysis support, manuscript editing, final approval of manuscript; A.K.: conception and design, financial support, project overseeing, manuscript writing, final approval of manuscript.

## Supporting information


**DATA S1**. Supplemental data

**FIGURE S1** Canine‐induced pluripotent stem cells shut down OKSM lentiviral transgene expression. qRT‐PCR showing repression of lentiviral transgenes in ciPSC after passage 15, when compared with a passage 4. Fold change expression of lentiviral cassette was assessed by primer pairs that bridge the OCT4 and KLF4, or KLF4 and SOX2 genes of the unique lentiviral transcriptional unit. Data represented is from n = 3 independent experiments, Mean ± SEM. Asterisk indicates significant difference with *P* < .01, when compared with ciPSC p4, by one‐way ANOVA.

**FIGURE S2** Pluripotency and differentiation marker expression in ciPSC and ciPSC‐derived differentiated EBs. qRT‐PCR showing downregulation of pluripotency markers and induction of differentiation markers upon differentiation of ciPSC. Normalization to undifferentiated ciPSC (ciPSC). EB, embryoid body differentiated ciPSC. n = 2, Mean ± SD. Asterisk indicates significance with *P* < .05, by Student t tests for each marker. Pluripotency markers: OCT4, SOX2, NANOG. Differentiation markers: KDR (mesoderm), AFP (endoderm), TUJ1 (ectoderm).

**FIGURE S3** OCT4‐eGFP reporter system construction. A, (i) Cas9‐sgRNA and (ii) cOCT4‐2A‐eGFP‐PGK‐Puro donor plasmid constructs. (iii) Schematic of the CRISPR/Cas9‐mediated editing of the canine OCT4 locus. cHA, canine homology arm. B, (i) Transfection efficiency shown as Relative mCherry Expression (count mCherry+ cells/DAPI nuclei) at day 2 PTr; and (ii) representative image of transfected CEF under puromycin selection at Day 5 PTr, showing expression of mCherry (red). C, Sequencing results of the carboxyterminal end of the canine endogenous OCT4 locus, showing insertion of the GFP sequence and downstream editions. Representative sequencing of edition results on two different CEF lines (CEF‐5 and CEF‐3). PTr, posttransfection.

**FIGURE S4** OKSIM transduction efficiency in adult stromal cells CDF and cASC. Infection evaluated by OCT4 immunofluorescence at 48/72 hours post‐transduction, defined as number of OCT4+ nuclei/number of DAPI+ nuclei. n = 10 for each group. Mean ± SEM.

**FIGURE S5** Population Doubling Time for CDF, cASC and CEF cell types. Population doubling time expressed in hours. Mean ± SD. NS, nonsignificant difference, as compared with CEF, *P* < .05, n = 4.

**FIGURE S6** Chromatin accessibility of pluripotency genes in stromal cells. Selected IGV genomic views of ATAC‐seq data for stemness genes *SOX2*, *NANOG*, *OCT4*, *KLF4*, and *MYC* for the three stromal cells CDF, cASC and CEF. All genome view vertical scales were group autoscaled to normalize for read‐depth. Genes are oriented 5′‐3′ and graphed from 2 kb upstream of the TSS to 2 kb downstream of the TES.

**FIGURE S7** Regulatory areas (promoter and 2 kb upstream and downstream), exons and introns are enriched in peaks with the highest fold‐change. Proportion of peaks over total peaks, found in different genomic areas when considering either all peaks in the data set or the highest FC peaks. FC, fold change.

**TABLE S1** Breed, age and sex of canine individuals per cell line.
**TABLE S2**. List of primers used for qRT‐PCR.
**TABLE S3**. List of antibodies used for immunofluorescence.

## Data Availability

The data that support the findings of this study are available from the corresponding author upon reasonable request.

## References

[1] Takahashi K , Tanabe K , Ohnuki M , et al. Induction of pluripotent stem cells from adult human fibroblasts by defined factors. Cell. 2007;131(5):861‐872. 18035408 10.1016/j.cell.2007.11.019

[2] Ezashi T , Yuan Y , Roberts RM . Pluripotent stem cells from domesticated mammals. Annu Rev Anim Biosci. 2016;4:223‐253. 26566158 10.1146/annurev-animal-021815-111202

[3] Brodehl A , Ebbinghaus H , Deutsch MA , et al. Human induced pluripotent stem‐cell‐derived cardiomyocytes as models for genetic cardiomyopathies. Int J Mol Sci. 2019;20(18):4381. 10.3390/ijms20184381 PMC6770343 31489928

[4] Lopes FM , Bristot IJ , da Motta LL , Parsons RB , Klamt F . Mimicking Parkinson's disease in a dish: merits and pitfalls of the most commonly used dopaminergic in vitro models. Neuromolecular Med. 2017;19(2–3):241‐255. 28721669 10.1007/s12017-017-8454-x

[5] Csobonyeiova M , Polak S , Danisovic L . Toxicity testing and drug screening using iPSC‐derived hepatocytes, cardiomyocytes, and neural cells. Can J Physiol Pharmacol. 2016;94(7):687‐694. 27128322 10.1139/cjpp-2015-0459

[6] Millman JR , Pagliuca FW . Autologous pluripotent stem cell‐derived beta‐like cells for diabetes cellular therapy. Diabetes. 2017;66(5):1111‐1120. 28507211 10.2337/db16-1406

[7] Mandai M , Watanabe A , Kurimoto Y , et al. Autologous induced stem‐cell‐derived retinal cells for macular degeneration. N Engl J Med. 2017;376(11):1038‐1046. 28296613 10.1056/NEJMoa1608368

[8] Yoshihara M , Hayashizaki Y , Murakawa Y . Genomic instability of iPSCs: challenges towards their clinical applications. Stem Cell Rev Rep. 2017;13(1):7‐16. 27592701 10.1007/s12015-016-9680-6 PMC5346115

[9] Natalwala A , Kunath T . Preparation, characterization, and banking of clinical‐grade cells for neural transplantation: scale up, fingerprinting, and genomic stability of stem cell lines. Prog Brain Res. 2017;230:133‐150. 28552226 10.1016/bs.pbr.2017.02.007

[10] Sasaki H , Wada H , Baghdadi M , et al. New immunosuppressive cell therapy to prolong survival of induced pluripotent stem cell‐derived allografts. Transplantation. 2015;99(11):2301‐2310. 26360665 10.1097/TP.0000000000000875

[11] Kol A , Arzi B , Athanasiou KA , et al. Companion animals: translational scientist's new best friends. Sci Transl Med. 2015;7(308):308‐321. 10.1126/scitranslmed.aaa9116 PMC4806851 26446953

[12] Bigi D , Marelli SP , Liotta L , et al. Investigating the population structure and genetic differentiation of livestock guard dog breeds. Animal. 2018;12(10):2009‐2016. 29331165 10.1017/S1751731117003573

[13] Hoffman JM , Creevy KE , Franks A , O'Neill DG , Promislow DEL . The companion dog as a model for human aging and mortality. Aging Cell. 2018;17(3):e12737. 29457329 10.1111/acel.12737 PMC5946068

[14] Hatoya S , Torii R , Kondo Y , et al. Isolation and characterization of embryonic stem‐like cells from canine blastocysts. Mol Reprod Dev. 2006;73(3):298‐305. 16292778 10.1002/mrd.20392

[15] Schneider MR , Adler H , Braun J , Kienzle B , Wolf E , Kolb HJ . Canine embryo‐derived stem cells—toward clinically relevant animal models for evaluating efficacy and safety of cell therapies. Stem Cells. 2007;25(7):1850‐1851. 17615273 10.1634/stemcells.2006-0357

[16] Hayes B , Fagerlie SR , Ramakrishnan A , et al. Derivation, characterization, and in vitro differentiation of canine embryonic stem cells. Stem Cells. 2008;26(2):465‐473. 18065395 10.1634/stemcells.2007-0640

[17] Vaags AK , Rosic‐Kablar S , Gartley CJ , et al. Derivation and characterization of canine embryonic stem cell lines with in vitro and in vivo differentiation potential. Stem Cells. 2009;27(2):329‐340. 19038794 10.1634/stemcells.2008-0433

[18] Wilcox JT , Semple E , Gartley C , et al. Characterization of canine embryonic stem cell lines derived from different niche microenvironments. Stem Cells Dev. 2009;18(8):1167‐1178. 19327015 10.1089/scd.2008.0336

[19] Tobias IC , Brooks CR , Teichroeb JH , Betts DH . Derivation and culture of canine embryonic stem cells. Methods Mol Biol. 2013;1074:69‐83. 23975806 10.1007/978-1-62703-628-3_6

[20] Shimada H , Nakada A , Hashimoto Y , Shigeno K , Shionoya Y , Nakamura T . Generation of canine induced pluripotent stem cells by retroviral transduction and chemical inhibitors. Mol Reprod Dev. 2010;77(1):2. 19890968 10.1002/mrd.21117

[21] Luo J , Suhr ST , Chang EA , et al. Generation of leukemia inhibitory factor and basic fibroblast growth factor‐dependent induced pluripotent stem cells from canine adult somatic cells. Stem Cells Dev. 2011;20(10):1669‐1678. 21495906 10.1089/scd.2011.0127 PMC3210032

[22] Lee AS , Xu D , Plews JR , et al. Preclinical derivation and imaging of autologously transplanted canine induced pluripotent stem cells. J Biol Chem. 2011;286(37):32697‐32704. 21719696 10.1074/jbc.M111.235739 PMC3173214

[23] Whitworth DJ , Ovchinnikov DA , Wolvetang EJ . Generation and characterization of LIF‐dependent canine induced pluripotent stem cells from adult dermal fibroblasts. Stem Cells Dev. 2012;21(12):2288‐2297. 22221227 10.1089/scd.2011.0608

[24] Chow L , Johnson V , Regan D , et al. Safety and immune regulatory properties of canine induced pluripotent stem cell‐derived mesenchymal stem cells. Stem Cell Res. 2017;25:221‐232. 29172152 10.1016/j.scr.2017.11.010 PMC6457437

[25] Goncalves NJN , Bressan FF , Roballo KCS , et al. Generation of LIF‐independent induced pluripotent stem cells from canine fetal fibroblasts. Theriogenology. 2017;92:75‐82. 28237347 10.1016/j.theriogenology.2017.01.013

[26] Nishimura T , Hatoya S , Kanegi R , et al. Feeder‐independent canine induced pluripotent stem cells maintained under serum‐free conditions. Mol Reproduct Develop. 2017;84(4):329‐339. 10.1002/mrd.22789 28240438

[27] Tsukamoto M , Nishimura T , Yodoe K , et al. Generation of footprint‐free canine induced pluripotent stem cells using auto‐erasable Sendai virus vector. Stem Cells Develop. 2018;27(22):1577‐1586. 10.1089/scd.2018.0084 30215317

[28] Baird A , Barsby T , Guest DJ . Derivation of canine induced pluripotent stem cells. Reproduct Domestic Anim Zuchthygiene. 2015;50(4):669‐676. 10.1111/rda.12562 26074059

[29] Ross PJ , Suhr ST , Rodriguez RM , et al. Human‐induced pluripotent stem cells produced under xeno‐free conditions. Stem Cells Devlop. 2010;19(8):1221‐1229. 10.1089/scd.2009.0459 20030562

[30] Luo J , Cibelli JB . Conserved role of bFGF and a divergent role of LIF for pluripotency maintenance and survival in canine pluripotent stem cells. Stem Cells Develop. 2016;25:1670‐1680. 10.1089/scd.2016.0164 27492281

[31] Li D , Liu J , Yang X , et al. Chromatin accessibility dynamics during iPSC reprogramming. Cell Stem Cell. 2017;21(6):819‐833 e816. 29220666 10.1016/j.stem.2017.10.012

[32] Buenrostro JD , Wu B , Chang HY , Greenleaf WJ . ATAC‐seq: a method for assaying chromatin accessibility genome‐wide. Curr Protocol Mol Biol. 2015;109:21 29 21‐21 29 29. 10.1002/0471142727.mb2129s109 PMC4374986 25559105

[33] Buenrostro JD , Giresi PG , Zaba LC , Chang HY , Greenleaf WJ . Transposition of native chromatin for fast and sensitive epigenomic profiling of open chromatin, DNA‐binding proteins and nucleosome position. Nat Methods. 2013;10(12):1213‐1218. 24097267 10.1038/nmeth.2688 PMC3959825

[34] Tripodi IJ , Allen MA , Dowell RD . Detecting differential transcription factor activity from ATAC‐Seq data. Molecules. 2018;23(5):1136. 10.3390/molecules23051136 PMC6099720 29748466

[35] Sipriani TM , Grandi F , da Silva LC , Maiorka PC , Vannucchi CI . Pulmonary maturation in canine foetuses from early pregnancy to parturition. Reprod Domest Anim. 2009;44(suppl 2):137‐140. 19754553 10.1111/j.1439-0531.2009.01446.x

[36] Kol A , Foutouhi S , Walker NJ , Kong NT , Weimer BC , Borjesson DL . Gastrointestinal microbes interact with canine adipose‐derived mesenchymal stem cells in vitro and enhance immunomodulatory functions. Stem Cells Devlop. 2014;23(16):1831‐1843. 10.1089/scd.2014.0128 PMC4120524 24803072

[37] GraphPad Software . GraphPad Prism v8. www.graphpad.com.

[38] Medvedev SP , Shevchenko AI , Elisaphenko EA , Nesterova TB , Brockdorff N , Zakian SM . Structure and expression pattern of Oct4 gene are conserved in vole *Microtus rossiaemeridionalis* . BMC Genomics. 2008;9:162. 18402712 10.1186/1471-2164-9-162 PMC2410140

[39] Gafni O , Weinberger L , Mansour AA , et al. Derivation of novel human ground state naive pluripotent stem cells. Nature. 2013;504(7479):282‐286. 24172903 10.1038/nature12745

[40] Lee BC , Kim MK , Jang G , et al. Dogs cloned from adult somatic cells. Nature. 2005;436(7051):641. 16079832 10.1038/436641a

[41] Parker HG , Kruglyak L , Ostrander EA . Molecular genetics: DNA analysis of a putative dog clone. Nature. 2006;440(7081):E1‐E2. 16525421 10.1038/nature04685 PMC3559127

[42] Garbutt TA , Konneker TI , Konganti K , et al. Permissiveness to form pluripotent stem cells may be an evolutionarily derived characteristic in *Mus musculus* . Sci Rep. 2018;8(1):14706. 30279419 10.1038/s41598-018-32116-8 PMC6168588

[43] Brons IG , Smithers LE , Trotter MW , et al. Derivation of pluripotent epiblast stem cells from mammalian embryos. Nature. 2007;448(7150):191‐195. 17597762 10.1038/nature05950

[44] Yeom YI , Fuhrmann G , Ovitt CE , et al. Germline regulatory element of Oct‐4 specific for the totipotent cycle of embryonal cells. Development. 1996;122(3):881‐894. 8631266 10.1242/dev.122.3.881

[45] Ye L , Zhang S , Greder L , et al. Effective cardiac myocyte differentiation of human induced pluripotent stem cells requires VEGF. PLoS One. 2013;8(1):e53764. 23326500 10.1371/journal.pone.0053764 PMC3542360

[46] Lian X , Bao X , Al‐Ahmad A , et al. Efficient differentiation of human pluripotent stem cells to endothelial progenitors via small‐molecule activation of WNT signaling. Stem Cell Rep. 2014;3(5):804‐816. 10.1016/j.stemcr.2014.09.005 PMC4235141 25418725

[47] Nazari B , Soleimani M , Ebrahimi‐Barough S , et al. Overexpression of miR‐219 promotes differentiation of human induced pluripotent stem cells into pre‐oligodendrocyte. J Chem Neuroanat. 2018;91:8‐16. 29530791 10.1016/j.jchemneu.2018.03.001

[48] Latchman DS , Brzeski H , Lovell‐Badge R , Evans MJ . Expression of the alpha‐fetoprotein gene in pluripotent and committed cells. Biochim Biophys Acta. 1984;783(2):130‐136. 6208940 10.1016/0167-4781(84)90004-6

[49] Nowicki M , Wierzbowska A , Malachowski R , et al. VEGF, ANGPT1, ANGPT2, and MMP‐9 expression in the autologous hematopoietic stem cell transplantation and its impact on the time to engraftment. Ann Hematol. 2017;96(12):2103‐2112. 28956132 10.1007/s00277-017-3133-4

[50] Huang J , Zhu H , Wang X , et al. The patterns and expression of KDR in normal tissues of human internal organs. J Mol Histol. 2011;42(6):597‐603. 21909756 10.1007/s10735-011-9355-1

[51] Pihlajamaa P , Sahu B , Janne OA . Determinants of receptor‐ and tissue‐specific actions in androgen signaling. Endocrine Rev. 2015;36(4):357‐384. 26052734 10.1210/er.2015-1034

[52] Jianmin W , Ruihua S , Lei C , et al. Construction of engineered murine embryonic stem cells with conditional knockout of FGFR2 depending on Cre‐loxP. Biocell. 2006;30(2):269‐278. 16972551

[53] Wamaitha SE , Grybel KJ , Alanis‐Lobato G , et al. IGF1‐mediated human embryonic stem cell self‐renewal recapitulates the embryonic niche. Nat Commun. 2020;11(1):764. 32034154 10.1038/s41467-020-14629-x PMC7005693

[54] Rocha CR , Lerner LK , Okamoto OK , Marchetto MC , Menck CF . The role of DNA repair in the pluripotency and differentiation of human stem cells. Mutat Res. 2013;752(1):25‐35. 23010441 10.1016/j.mrrev.2012.09.001

[55] Ladstatter S , Tachibana‐Konwalski K . A surveillance mechanism ensures repair of DNA lesions during zygotic reprogramming. Cell. 2016;167(7):1774‐1787. e1713. 27916276 10.1016/j.cell.2016.11.009 PMC5161750

[56] Bigarella CL , Liang R , Ghaffari S . Stem cells and the impact of ROS signaling. Development. 2014;141(22):4206‐4218. 25371358 10.1242/dev.107086 PMC4302918

[57] Wong SY , Soto J , Li S . Biophysical regulation of cell reprogramming. Curr Opin Chem Eng. 2017;15:95‐101. 28413770 10.1016/j.coche.2017.01.001 PMC5390558

[58] Iwafuchi‐Doi M , Zaret KS . Pioneer transcription factors in cell reprogramming. Genes Develop. 2014;28(24):2679‐2692. 25512556 10.1101/gad.253443.114 PMC4265672

[59] Aydin B , Mazzoni EO . Cell reprogramming: the many roads to success. Annu Rev Cell Develop Biol. 2019;35:433‐452. 10.1146/annurev-cellbio-100818-125127 31340126

[60] Gilbert LA , Larson MH , Morsut L , et al. CRISPR‐mediated modular RNA‐guided regulation of transcription in eukaryotes. Cell. 2013;154(2):442‐451. 23849981 10.1016/j.cell.2013.06.044 PMC3770145

[61] Prasad M , Kumar B , Bhat‐Nakshatri P , et al. Dual TGFbeta/BMP pathway inhibition enables expansion and characterization of multiple epithelial cell types of the normal and cancerous breast. Mol Cancer Res. 2019;17(7):1556‐1570. 30992305 10.1158/1541-7786.MCR-19-0165 PMC6610652

[62] Zhang QZ . Dystroglycan induced muscular dystrophies ‐ a review. Eur Rev Med Pharmacol Sci. 2016;20(17):3683‐3687. 27649671

[63] Huang HP , Chen PH , Yu CY , et al. Epithelial cell adhesion molecule (EpCAM) complex proteins promote transcription factor‐mediated pluripotency reprogramming. J Biol Chem. 2011;286(38):33520‐33532. 21799003 10.1074/jbc.M111.256164 PMC3190890

[64] Megyola CM , Gao Y , Teixeira AM , et al. Dynamic migration and cell‐cell interactions of early reprogramming revealed by high‐resolution time‐lapse imaging. Stem Cells. 2013;31(5):895‐905. 23335078 10.1002/stem.1323 PMC4309553

[65] Pieters T , van Roy F . Role of cell‐cell adhesion complexes in embryonic stem cell biology. J Cell Sci. 2014;127(Pt 12):2603‐2613. 24931943 10.1242/jcs.146720

[66] Hawkins K , Joy S , McKay T . Cell signalling pathways underlying induced pluripotent stem cell reprogramming. World J Stem Cells. 2014;6(5):620‐628. 25426259 10.4252/wjsc.v6.i5.620 PMC4178262

[67] Cheng Y , Cheung AK , Ko JM , et al. Physiological beta‐catenin signaling controls self‐renewal networks and generation of stem‐like cells from nasopharyngeal carcinoma. BMC Cell Biol. 2013;14:44. 24073846 10.1186/1471-2121-14-44 PMC3819748

[68] Martin‐Lopez M , Maeso‐Alonso L , Fuertes‐Alvarez S , et al. p73 is required for appropriate BMP‐induced mesenchymal‐to‐epithelial transition during somatic cell reprogramming. Cell Death Disease. 2017;8(9):e3034. 28880267 10.1038/cddis.2017.432 PMC5636977

[69] Karin M , Liu Z , Zandi E . AP‐1 function and regulation. Curr Opin Cell Biol. 1997;9(2):240‐246. 9069263 10.1016/s0955-0674(97)80068-3

[70] Angel P , Karin M . The role of Jun, Fos and the AP‐1 complex in cell‐proliferation and transformation. Biochim Biophys Acta. 1991;1072(2–3):129‐157. 1751545 10.1016/0304-419x(91)90011-9

[71] Liu J , Han QK , Peng TR , et al. The oncogene c‐Jun impedes somatic cell reprogramming. Nat Cell Biol. 2015;17(7):856‐867. 26098572 10.1038/ncb3193

[72] Maruyama M , Ichisaka T , Nakagawa M , Yamanaka S . Differential roles for Sox15 and Sox2 in transcriptional control in mouse embryonic stem cells. J Biol Chem. 2005;280(26):24371‐24379. 15863505 10.1074/jbc.M501423200

[73] Sazonova O , Zhao Y , Nurnberg S , et al. Characterization of TCF21 downstream target regions identifies a transcriptional network linking multiple independent coronary artery disease loci. PLoS Genet. 2015;11(5):e1005202. 26020271 10.1371/journal.pgen.1005202 PMC4447360

[74] Grskovic M , Chaivorapol C , Gaspar‐Maia A , Li H , Ramalho‐Santos M . Systematic identification of cis‐regulatory sequences active in mouse and human embryonic stem cells. PLoS Genet. 2007;3(8):e145. 17784790 10.1371/journal.pgen.0030145 PMC1959362

[75] Jang J , Wang Y , Kim HS , Lalli MA , Kosik KS . Nrf2, a regulator of the proteasome, controls self‐renewal and pluripotency in human embryonic stem cells. Stem Cells. 2014;32(10):2616‐2625. 24895273 10.1002/stem.1764 PMC4165656

[76] Megquier K , Genereux DP , Hekman J , et al. BarkBase: Epigenomic annotation of canine genomes. Genes. 2019;10(6):433. 10.3390/genes10060433 PMC6627511 31181663

