# Supplementary material for: Chromatin accessibility in canine stromal cells and its implications for canine somatic cell reprogramming
Source: Stem Cells Transl Med. 2020 Nov 16;10(3):441–54. doi: 10.1002/sctm.20-0278 (PMC7900587; doi:10.1002/sctm.20-0278)
Supplement: Supplementary file 1 — DATA S1. Supplemental data [file SCT3-10-441-s001.docx]

**Supplemental Materials and Methods**

Population Doubling Time

Cells from a passage 2 were counted and 50,000 cells each of CDF, cASC or CEF were plated in each of a 6 well plate well. Cells were harvested by trypsinization with 0.05% Trypsin/EDTA at 37°C for 5 minutes, and counted for 5 consecutive days, every 24 hours. The results were graphed as cell number vs. Day, and an exponential curve trendline was calculated. The linear part of the exponential curve was determined to be between Day 2 and Day 3, and was used to calculate Population Doubling Times (PDT), considering an initial lag phase of 24 hours [^1^](#_ENREF_1).

$$PDT=\frac{Duration \left( days \right)\times log(2)}{\log\left( Final Cell Number \right)-\log(Initial Cell Number)}$$

ANOVA was used to study statistical differences, p<0.05 n=4.

Generation of OCT4-eGFP cells

We sought to create a live-cell OCT4 fluorescent reporter system by tagging the native OCT4 locus with the enhanced green fluorescent protein (eGFP), such that native OCT4 expression will be coupled with eGFP expression. A cassette containing a 2A cleavage site, followed by eGFP, the phosphoglycerate kinase (PGK) constitutive promoter controlling the expression of the Puromycin resistance gene was inserted in tandem with the native canine *OCT4* genomic locus in CEF. Gene editing was achieved using the CRISPR/Cas9 homology directed repair (HDR) method, utilizing a D10A Cas9 nickase and 2 gRNA in order to diminish off-target effects and increase sensitivity, and donor DNA containing the transgenes flanked by homology arms contiguous to the site of insertion, at the carboxyterminal end of the OCT4 locus. Briefly, we generated a donor construct carrying a 2A-eGFP-PGK-Puro cassette flanked by homology arms (HA) for the carboxyterminal end of the canine *OCT4* gene. To this aim, the canine HAs (cHAs) for the *OCT4* gene were obtained from CEF by PCR amplification of the end of Exon 5, and were cloned into the OCT4-2A-eGFP-PGK-Puro plasmid, which was a gift from Rudolf Jaenisch (Addgene plasmid #31938) [^2^](#_ENREF_2), after removal of the human sequences for *OCT4*, by restriction enzyme digestion and T4 ligase ligation (New England BioLabs). The left cHA1 comprised the 700 bp sequence upstream of the Transcription End Site (TES) of *OCT4*, and the right cHA2 comprised the 700 bp after said site. sgRNA sequences were designed on CHOPCHOP v3 [^3^](#_ENREF_3), and cloned into the CRISPR/Cas9 AIO-mCherry plasmid, which was a gift from Steve Jackson (Addgene plasmid #74120) [^4^](#_ENREF_4), by Golden Gate cloning. The sgRNA1 (antisense) sequence was GGAAGAAGGAGACCGGGGGTAAG and the sgRNA2 (sense) sequence was TCGTGGAGTTAAACTTACGTACC. The edition included stop codon mutation to a neutral small amino acid, double PAM mutation and the insertion of the -2A-GFP/PGK-Puromycin cassette after the endogenous OCT4 locus. The two constructs generated finally generated for the editing contained i) the Cas9-mCherry fusion protein under a constitutive promoter and the U6 promoter controlling the expression of 2 sgRNAs, and ii) eGFP donor DNA flanked by OCT4 homology arms (Supplemental Figure S3A). Both plasmids were co-transfected into CEF and mCherry expression was assessed starting 48 hours post-transfection (PTr) with over 20% efficiency, indicating successful transfection and expression of Cas9 (Supplemental Figure S3B). Sanger sequencing after puromycin selection of the transgenic CEF confirmed appropriate genomic integration (Supplemental Figure S3C). Transgenic CEF did not show any eGFP expression prior to OKSIM transduction. Sequencing of the constructs was performed at the UCD College of Biological Sciences sequencing core with an ABI Prism® 3730 Genetic Analyzer and ABI Prism® 3730 Data Collection Software v. 3.0, and the Sanger sequencing service at GeneWiz. Sequencing results were visualized and captured with Benchling [^5^](#_ENREF_5).

CEF cells were co-transfected with the donor and CRISPR/Cas9 plasmids with Lipofectamine 3000 (Thermo Fisher). Shortly, 150,000 cells were seeded in 6 well multiwell plates and transfected 24 hours later, according to manufacturer’s instructions. mCherry expression was followed starting 48 hours PTr. Puromycin treatment at 1.5 ng/ml was started at 48 hours PTr and continued until day 14 PTr, on which cells were passed and expanded for 48 hours without antibiotic. Genomic eGFP insertion after the *OCT4* coding sequence in CEF was confirmed by sequencing. 48 hours after the passage, cells were reprogrammed as described in “Generation of canine induced pluripotent stem cells” below. Green colonies were observed starting at Day 14 post- transduction (PT).

Lentivirus production

HEK-293T cells were plated the day before for 70-90% confluence the next day. Plasmid DNA was transfected into HEK-293T cells with Fugene 6 (Roche), according to manufacturer’s instructions. Briefly, DNA was mixed in a Transfer:Tat/Rev/Gag-Pol (psPAX2):VSV-G (pMD2.G) 10:6.5:1.5 ratio and combined with Fugene 6 at a Fugene 6: total DNA ratio of 3:1. The DNA-Fugene 6 mix was added to the cells, and lentiviral supernatant was collected 48 hours PT, and filtered through a 0.45 µm low-binding PVDF filter. The transfer plasmid used was OKSIM [^6^](#_ENREF_6), which was a gift from José Cibelli (Addgene plasmid # 24603). The lentiviral vector generated from OKSIM carries the human OSKM genes under a constitutive EF1α promoter, as previously described [^6^](#_ENREF_6).

Generation of canine induced pluripotent stem cells

100,000 CEF at passage 1-4 in a 6-well plate were transduced with fresh OKSIM lentiviral media collected on the same day and 25% fresh complete DMEM media, for a final volume of 2 ml with 10 µg/ml Polybrene (Millipore) per well. On day 2 PT, media was replaced with ciPSC media comprised of DMEM/F12 supplemented with 20% Knockout Serum Replacement (KSR), 0.1-mM NEAA, 2-mM GlutaMax, Pen/Strep, 0.075 mM β-mercaptoethanol (Sigma-Aldrich), 8 ng/ml bFGF and 10-ng/ml of LIF (Peprotech), and on day 4 PT cells were dissociated with TRypLE Express (Gibco) and plated in 10 cm plates with fresh iMEF at 100-200,000 CEF cells per plate. On day 5-10 PT, ciPSC colonies became visible as tight cell aggregates with prominent nucleoli, phase-bright perinuclear material, and high nucleus:cytoplasm ratio. Depending on the size of the colonies, these were picked between days 14 and 21 PT. The first 2-3 passages were performed manually, with each colony being transferred to an individual well of a 12-well plate with fresh iMEF. After 3 passages, clones were considered established ciPSC lines and passaged by dissociation with 1 mg/ml collagenase type IV (Gibco) at 37°C for 30 minutes, and plated onto fresh iMEF feeder layers, every 3-5 days. To induce differentiation, ciPSC and cESC colonies were dispersed with 1 mg/ml collagenase type IV, and then transferred to ultra-low attachment plates (Corning) in DMEM media, consisting of DMEM supplemented with 20% FBS, 0.1 mM NEAA, 2 mM GlutaMax, 1 mM sodium pyruvate, Pen/Strep. Cells were incubated in suspension for 7 days, during which they aggregated to form embryoid bodies (EB), which were then plated on 0.1% bovine gelatin (Sigma-Aldrich)-coated 24-well plates and cultured for an additional 7-14 days.

Alkaline phosphatase detection

Alkaline phosphatase (AP) activity was detected using the Alkaline Phosphatase Staining Kit II (StemGent), according to manufacturer’s instructions. Briefly, cells were fixed and subsequently incubated with the AP substrate solution, at room temperature in the dark for 5-15 minutes. The reaction was stopped by aspirating the AP substrate solution and washing the wells. AP expression resulted in a red stain, while the absence of AP expression resulted in no stain. Cells were covered with PBS and images were obtained in a FluorChem E (Protein Simple) imaging system.

Vector construction for OCT4 enhancer analysis

*OCT4* enhancer regions Distant Enhancer (DE) and Proximal Enhancer (PE), as previously described ^[7](#_ENREF_7)^, were obtained from CEF cells by PCR amplification, and cloned by restriction enzyme digestion and ligase ligation, individually into the firefly luciferase (luc) reporter vector, pGL3-Promoter (Promega). This plasmid contains an SV40 minimal promoter upstream of the luc gene, and multiple cloning sites (MCS) allowing for DNA fragments containing putative enhancer elements to be inserted upstream or downstream of the promoter. The pGL3-Control vector (Promega), which contains both the SV40 promoter and enhancer sequences, resulting in strong expression of luc, was used as a control for monitoring transfection efficiency, and as an internal standard for promoter and enhancer activity. The pIS2 plasmid, a gift from David Bartel (Addgene #12177) [^8^](#_ENREF_8) was used for normalization; pIS2 contains the Renilla luciferase reporter driven by the SV40 early enhancer and promoter. Sequencing was used to check all cloning, as described above. The obtained DE and PE sequences were the following:

OCT4 DE: ACTGGCATGGGGAGAGAGGGTTCTGGGAGCGTAGAATACGCTTTCTGGGAAGCAATGGTGTAGGGGATTTCAGCCAAGACCTAGGGCTGCCCTTCCCCCTCCTCCAGGAGGCTGCCTTCATGGCAGACAGCAGATAGATGCATGACAAAGGTGCCGTGATGGCCCTGTCCCGGGGCGGGGGGGGGGGGGGGGGCCAAAATTGCTTGGGAGGGGCCCCCTCCTGTTCTAATGCTCGTCTCTCCACCCCCACCTGGCCTCTTAATCTATCTGCCTTTTGGGCAGTTAGTGG

OCT4 PE: ACCCTGGGCGGGAGAGTTTAGGGCAGGCTTCCTGCACCCCCTCCCCAAATCGCTCTCCACCTCTTCAGCTTCTTTCAACCAGCCCCACTAAACAAAGTGCATCCCTTGGCCTGGGGCTCTGGGGGAGGGGTGGATGAGGAGGCTGGACGCCCCAGTCCTCCAGAGGAAGGGGAGCAGGATACCTAGGTTCTCAATGGGGGGCCCCGTCTGAGGCTCAGGCTTTGAGGGGATAGGGGGGTGGTGTTGCTGGAGTTCTTTTAGCTGCTTTGAGGGGGATTCTGTGGGGGGGGGATTGGGGCTGGGGGTTCAAAGGCAATGTTTTATACAAAACATGTAGATAAAACCAAAAGGCCTCAAATAGAAGTGAA

Nucleofection of cPSCs and Luciferase Assays

DNA Constructs were electroporated into ciPSC with an Amaxa Nucleofector 2b Device (Lonza), using the Amaxa Human Stem Cell Nucleofector Kit 1 (Lonza). Briefly, ciPSC were harvested by collagenase type IV incubation for 1 hour at 37°C, and mechanically dissociated to single cell by up and down pipetting. 500,000 cells were resuspended in Nucleofector solution 1 + plasmid DNA solution in a nucleofection cuvette, electroporated using program A-023, and re-plated on iMEF feeder layers. Firefly and Renilla Luciferase activity was assayed with the Dual-Glo Luciferase Assay System (Promega) following manufacturer’s instructions, and bioluminescence results were read in a Veritas Microplate Luminometer (Turner Biosystems) using Veritas (Turner Biosystems) software. The ratio of Firefly luciferase over Renilla luciferase luminescence was normalized to the same ratio of pGL3-Control control wells. Data was analyzed for significance by One-Way ANOVA and Friedman multiple comparisons tests.

RNA extraction, cDNA synthesis and RT-Quantitative PCR (RT-qPCR)

Total RNA was extracted with the RNeasy Mini Kit (Qiagen), as recommended by the manufacturer with a DNAse I incubation step. cDNA was synthesized from 1000 ng of total RNA with the First-strand cDNA Synthesis for Quantitative RT-PCR (OriGene), according to manufacturer’s instructions. The cDNA samples were diluted 10-fold for qPCR reactions.

PCR amplification was performed in a StepOnePlus Real-Time PCR System (Applied Biosystems), using PowerUp SYBR Green Master Mix (Applied Biosystems) with fast settings at an annealing temperature of 60°C. Canine primer design was performed on NCBI’s Primer Blast, and primer validation included a melting curve analysis, amplicon size confirmation by gel electrophoresis, and amplicon sequence confirmation by band sequencing. qPCR analysis was performed on the StepOne Software v2. 1, with canine GAPDH as an endogenous control.

To assess expression of the OKSIM transgenes, RT-qPCR was performed on cDNA from the ciPSC being assessed, CEF and a low passage (p4) of ciPSC, using primers that bridge over two of the four transgenes on the vector sequence. The transgene cassette is expressed as a single mRNA transcriptional unit. This design allows for ampliﬁcation of only cDNA generated from exogenous mRNA expression of these genes[^6^](#_ENREF_6). The primers bridge over OCT4 and KLF4 (OK), or over KLF4 and SOX2 (KS). Primer OK was designed by Luo et al [^9^](#_ENREF_9), and Primers KS were designed for this study.

Primers used can be found in Supplemental Table S2.

Immunofluorescence

Briefly, cells were washed with PBS and fixed in 4% formaldehyde (PFA), permeabilized and blocked with PBS-0.1% Albumin (PBSA), 0.1% Triton X-100, 10% normal donkey serum (NDS), and stained with primary antibodies at an appropriate dilution in PBSA 10% NDS. Cells were then stained with fluorescent secondary antibodies (Invitrogen) diluted in PBSA 10% NDS at 1:200, and nuclei stained with DAPI (Sigma-Aldrich). Stained cells were viewed, and images were captured, with an Evos FL digital inverted epifluorescence imaging system (Life Technologies). Antibodies used can be found in Supplemental Table S3.

iMEF depletion

In order to obtain pure samples without iMEF contamination for ATAC-seq library preparation, ciPSC and cESC samples were iMEF depleted using MACS Feeder Removal MicroBeads (FRMs, Miltenyi Biotec). ciPSC in feeder layers were harvested by incubation in Collagenase type IV for 1 hour at 37°C, followed by incubation in Trypsin for 5 min at 37°C. The cell mix suspension was then passed through a 30 μm Pre-Separation Filter (Miltenyi Biotec) and magnetically labeled with the FRMs conjugated to monoclonal anti‑mouse feeder antibodies, by incubation of 10^7^ cells in 80 μl culture medium + 20 μl FRMs for 15 minutes at 4°C. Next, the cell suspension was loaded onto a MACS LS Column and placed in the magnetic field of a MACS Separator (all Miltenyi Biotec), according to manufacturer recommendations. The magnetically labeled fibroblasts are retained within the column, while the unlabeled ciPSC are recovered in the flow-through.

ATAC-seq chromatin accessibility assay

The assay was carried out as previously described [^10^](#_ENREF_10). Briefly, open chromatin DNA libraries were prepared from a crude nuclei extract from 50,000 cells of each cell sample, by lysis with cold lysis buffer, consisting of 10 mM Tris-HCl, pH 7.4, 10 mM NaCl, 3 mM MgCl2 and 0.2% IGEPAL CA-630. Cells growing on iMEF feeder layers were iMEF depleted, as described above. Immediately after lysis, nuclei were spun and resususpended in the transposase reaction mix, consisting of 25 μL TD buffer, 2.5 μL transposase (Illumina) and 22.5 μL nuclease-free water, per reaction. The transposition reaction was carried out for 60 min at 37 °C, with agitation. Directly following transposition the sample was purified using a MinElute column (Qiagen), and the library was amplified with SsoFast EvaGreen Supermix (Bio-Rad Laboratories) and Nextera PCR primers [5], using the following PCR conditions: 72°C for 5 min; 98°C for 30 s; and thermocycling at 98°C for 10 s, 63°C for 30 s and 72°C for 1 minute. To reduce GC and size bias we monitored the PCR reaction using qPCR in order to stop amplification before saturation, according to [^10^](#_ENREF_10). To do this, we amplified the full libraries for five cycles, after which we took a 5 μl aliquot of the PCR reaction and added 10 μl of PowerUp SYBR Green Master Mix and Nextera primers, at a final concentration of 0.6×. We ran this reaction for 40 more cycles to determine the additional number of cycles needed for the remaining 45 μL reaction, calculated as 1/3 or the maximum Rn. Libraries were amplified for a total of 15–21 cycles purified using Agencourt AMPure XP beads (Beckman Coulter), and eluted in 40 μl buffer EB. Libraries were quality controlled and quantified in a BioAnalyzer 2100 (Agilent Technologies), and sequenced in an Illumina HiSeq4000 system in a paired-end 150 bp run; at least 50,000,000 raw reads per sample were obtained, ultimately yielding a minimum of 14,000,000 non mithocondrial, cleaned, mapped reads per cell line. In all cases 3 biological replicates per cell line, and 4 cell lines per cell type were used, except for cESC, for which we only had 1 cell line available.

ATAC-seq data analysis

We used the HTStream [^11^](#_ENREF_11) for data pre-processing (cleaning, duplicate removal, adapter and quality trimming, and pair-ended reads overlapping). Only unique reads mapping to a single genomic location and strand were kept. All sequencing data was mapped to the CanFam3.1 canine genome assembly using Burrows-Wheeler Alignment (BWA) [^12^](#_ENREF_12) tools for indexing and alignment, and SAMtools [^13^](#_ENREF_13) for filtering, sorting, and mitochondrial removal using ‘grep -v MT’. Biological replicates were merged and macs2 [^14^](#_ENREF_14) was used for ATAC-seq broad peak calling. Quality control was performed in R [^15^](#_ENREF_15), using the packages knitr [^16^](#_ENREF_16), kableExtra [^17^](#_ENREF_17), jsonlite [^18^](#_ENREF_18), readr [^19^](#_ENREF_19), and stringr [^20^](#_ENREF_20). BigWig files were produced using bamCoverageBed with CPM normalization from deeptools [^21^](#_ENREF_21), and npz matrix files were produced using multiBigwigSummary from deeptools, for PCA and Spearman correlation plots, representing the correlation strength between paired samples. Deeptools heatmaps show genes from TSS to TES representing the same size for the length of every gene, as is common practice in the field [^22^](#_ENREF_22). Next, replicate peaks were consolidated into one individual peak as previously described [^23^](#_ENREF_23), followed by pairwise overlaps per cell type, finally creating a table of peaks vs. cell type, where if there is an overlap between the peak and the cell type the value is the read depth at the peak summit, and if there is no overlap the value is zero. Statistical analysis of differential chromatin openness on the aforementioned tables was conducted using the limma-voom Bioconductor pipeline [^24^](#_ENREF_24) (using edgeR version 3.20.9, limma version 3.34.9, in R 3.4.4). Normalization for differences in total read count was conducted using TMM normalization [^25^](#_ENREF_25), and peaks were annotated using the Bioconductor package ChIPseeker, version 1.20.0 [^26^](#_ENREF_26) and the Bioconductor database TxDb.Cfamiliaris.UCSC.canFam3.refGene, version 3.4.6, in R 3.6.0. Gene names and descriptions from Ensembl were then added. Venn diagrams were constructed in R with the VennDiagram [^27^](#_ENREF_27) and venn [^28^](#_ENREF_28) libraries. Read depth heatmaps and profile plots were generated using deeptools. ATAC-seq profiles were also represented by IGV v2.7.2 software [^29^](#_ENREF_29). Hierarchical clustering was performed by Pearson correlation distance calculation with Cluster 3.0 [^30^](#_ENREF_30) and visualization with Java TreeView [^31^](#_ENREF_31). In the case of Figure 2E, the data was filtered to construct the heat map: starting with a set comprised of all peaks, the dataset was filtered, removing peaks when not one of the samples had more than 1.1 normalized reads and when the samples with the highest and lowest expression were less than 1.5-fold different In the rest of the hierarchical clustering heat maps, data is the full dataset for the comparison at hand in each case. Data was organized by uncentered Pearson correlation into a matrix and hierarchical clustering by peak and sample. Clusters were defined visually by peak clustering patterns. Gene Ontology (GO) term classification was performed with PANTHER [^32^](#_ENREF_32). GO term enrichment analysis was performed within the “Biological Process”, “Pathway” and “Protein Class” main GO classifications. The data was analyzed on the PANTHER database by the PANTHER Overrepresentation Test (Released 20200728), PANTHER version 15.0 Released 2020-02-14, using a reference list of all genes in the *Canis lupus familiaris* database as background. We used Fisher's Exact test with a Binomial correction to calculate False Discovery Rate (FDR), with p< 0.05, FDR < 0.05, and Fold Enrichment > 2. Identification of candidate barrier genes was performed by filtering of genes by GO term in the PANTHER database. Genes classified under GO term “Biological Process” and sub-GO terms “Biological Adhesion”, “Reproduction”, “Reproductive Process”, “Developmental Process”, “Metabolic Process” “Response to Stimulus”, and “Signaling“ and GO term “Protein Class”, sub-GO terms “Cell Adhesion Molecule”, “ TF”, “Gene Specific Transcriptional Regulator”, “Intercellular Signal Molecule”, “Nucleic Acid Binding Protein”, “Transmembrane Signal Receptor”, and “Receptor” were kept for further analysis, since these are the most relevant classifications.

TF motif enrichment was examined in the peaks that reached statistical significance (adjusted p-value < 0.05) between two conditions at a time. These peaks were further separated into two groups, with one group corresponding to the peaks having higher DNA occupancy in condition one and the second group corresponding to the peaks having higher occupancy in condition two. The peaks that did not show significant difference between the two conditions were used as background in an enrichment analysis performed with HOMER [^33^](#_ENREF_33), running findMotifs.pl with FASTA files (-fasta). Enriched motifs found were only kept if the P value was < 1x10^-50^ and Fold Induction (percent of target/percent of background) was > 1.5. Figures show up to 15 motifs sorted by Fold Induction in descending order.

Use of free license images

We used the Library of Science & Medical Illustrations[^34^](#_ENREF_34) and the SMART Servier Medical Art libraries[^35^](#_ENREF_35) for figure construction.

**Supplemental References**

1 Daukste L, Basse B, Baguley BC, Wall DJ. Mathematical determination of cell population doubling times for multiple cell lines. Bull Math Biol*.* 2012;74(10):2510-2534.

2 Hockemeyer D, Wang H, Kiani S, et al. Genetic engineering of human pluripotent cells using TALE nucleases. Nat Biotechnol*.* 2011;29(8):731-734.

3 Labun K, Montague TG, Krause M, Torres Cleuren YN, Tjeldnes H, Valen E. CHOPCHOP v3: expanding the CRISPR web toolbox beyond genome editing. Nucleic Acids Res*.* 2019;47(W1):W171-W174.

4 Chiang TW, le Sage C, Larrieu D, Demir M, Jackson SP. CRISPR-Cas9(D10A) nickase-based genotypic and phenotypic screening to enhance genome editing. Sci Rep*.* 2016;6:24356.

5 Benchling Computer Software. Benchling [Biology Software]. (2019). Retrieved 2019, December 19 from https://benchling.com.

6 Ross PJ, Suhr ST, Rodriguez RM, et al. Human-induced pluripotent stem cells produced under xeno-free conditions. Stem cells and development*.* 2010;19(8):1221-1229.

7 Medvedev SP, Shevchenko AI, Elisaphenko EA, Nesterova TB, Brockdorff N, Zakian SM. Structure and expression pattern of Oct4 gene are conserved in vole Microtus rossiaemeridionalis. BMC genomics*.* 2008;9:162.

8 Farh KK, Grimson A, Jan C, et al. The widespread impact of mammalian MicroRNAs on mRNA repression and evolution. Science*.* 2005;310(5755):1817-1821.

9 Luo J, Suhr ST, Chang EA, et al. Generation of leukemia inhibitory factor and basic fibroblast growth factor-dependent induced pluripotent stem cells from canine adult somatic cells. Stem Cells Dev*.* 2011;20(10):1669-1678.

10 Buenrostro JD, Wu B, Chang HY, Greenleaf WJ. ATAC-seq: A Method for Assaying Chromatin Accessibility Genome-Wide. Current protocols in molecular biology*.* 2015;109:21 29 21-21 29 29.

11 Institute for Bioinformatics and Evolutionary Studies. HTStream. A toolset for high throughput sequence analysis using a streaming approach facilitated by Linux pipes. Retrieved 2019, October 25 from https://ibest.github.io/HTStream/.

12 Li H, Durbin R. Fast and accurate short read alignment with Burrows-Wheeler transform. Bioinformatics*.* 2009;25(14):1754-1760.

13 Li H, Handsaker B, Wysoker A, et al. The Sequence Alignment/Map format and SAMtools. Bioinformatics*.* 2009;25(16):2078-2079.

14 Thomas R, Thomas S, Holloway AK, Pollard KS. Features that define the best ChIP-seq peak calling algorithms. Brief Bioinform*.* 2017;18(3):441-450.

15 R Core Team. R: A language and environment for statistical computing. R Foundation for Statistical Computing, Vienna, Austria. URL https://[www.R-project.org/](http://www.R-project.org/). 2019.

16 Xie Y. knitr: A Comprehensive Tool for Reproducible Research in R. In Victoria Stodden, Friedrich Leisch and Roger D. Peng, editors, Implementing Reproducible Computational Research. Chapman and Hall/CRC. ISBN 978-1466561595. 2014.

17 Zhu H. kableExtra: Construct Complex Table with 'kable' and Pipe Syntax. R package version 1.1.0. https://CRAN.R-project.org/package=kableExtra. 2019.

18 Ooms J. The jsonlite Package: A Practical and Consistent Mapping Between JSON Data and R Objects. arXiv:1403.2805 [stat.CO] URL https://arxiv.org/abs/1403.2805. 2014.

19 Wickham H, Hester J, Francois R, R Core Team. readr: Read Rectangular Text Data. R package version 1.3.1. https://CRAN.R-project.org/package=readr. 2018.

20 Wickham H. stringr: Simple, Consistent Wrappers for Common String Operations. R package version 1.4.0. https://CRAN.R-project.org/package=stringr. 2019.

21 Ramirez F, Dundar F, Diehl S, Gruning BA, Manke T. deepTools: a flexible platform for exploring deep-sequencing data. Nucleic acids research*.* 2014;42(Web Server issue):W187-191.

22 Li D, Liu J, Yang X, et al. Chromatin Accessibility Dynamics during iPSC Reprogramming. Cell stem cell*.* 2017;21(6):819-833 e816.

23 Wang J, Zibetti C, Shang P, et al. ATAC-Seq analysis reveals a widespread decrease of chromatin accessibility in age-related macular degeneration. Nature communications*.* 2018;9(1):1364.

24 Ritchie ME, Phipson B, Wu D, et al. limma powers differential expression analyses for RNA-sequencing and microarray studies. Nucleic Acids Res*.* 2015;43(7):e47.

25 Robinson MD, Oshlack A. A scaling normalization method for differential expression analysis of RNA-seq data. Genome Biol*.* 2010;11(3):R25.

26 Yu G, Wang LG, He QY. ChIPseeker: an R/Bioconductor package for ChIP peak annotation, comparison and visualization. Bioinformatics*.* 2015;31(14):2382-2383.

27 Chen H. VennDiagram: Generate High-Resolution Venn and Euler Plots. R package version 1.6.20. https://CRAN.R-project.org/package=VennDiagram. 2018.

28 Dusa A. venn: Draw Venn Diagrams. R package version 1.8. https://CRAN.R-project.org/package=venn. 2019.

29 Robinson JT, Thorvaldsdottir H, Winckler W, et al. Integrative genomics viewer. Nature biotechnology*.* 2011;29(1):24-26.

30 de Hoon MJ, Imoto S, Nolan J, Miyano S. Open source clustering software. Bioinformatics*.* 2004;20(9):1453-1454.

31 Saldanha AJ. Java Treeview--extensible visualization of microarray data. Bioinformatics*.* 2004;20(17):3246-3248.

32 Mi H, Dong Q, Muruganujan A, Gaudet P, Lewis S, Thomas PD. PANTHER version 7: improved phylogenetic trees, orthologs and collaboration with the Gene Ontology Consortium. Nucleic acids research*.* 2010;38(Database issue):D204-210.

33 Heinz S, Benner C, Spann N, et al. Simple combinations of lineage-determining transcription factors prime cis-regulatory elements required for macrophage and B cell identities. Mol Cell*.* 2010;38(4):576-589.

34 Created by somersault18:24. <http://www.somersault1824.com>. Accessed on June 25, 2020. Creative Common License Attribution-NonCommercial-ShareAlike 4.0 International (CC BY-NC-SA 4.0).

35 Created by Servier. https://smart.servier.com/. Accessed on June 25, 2020. Creative Common License Attribution 3.0 Unported (CC BY 3.0)

**Supplemental Figure Legends**

**Supplemental Figure S1. Canine induced Pluripotent Stem Cells shut down OKSM lentiviral transgene expression.**

qRT-PCR showing repression of lentiviral transgenes in ciPSC after passage 15, when compared to a passage 4. Fold Change expression of lentiviral cassette was assessed by primer pairs that bridge the OCT4 and KLF4, or KLF4 and SOX2 genes of the unique lentiviral transcriptional unit. Data represented is from n = 3 independent experiments, Mean ± SEM. * indicates significant difference with p < 0.01, when compared to ciPSC p4, by One-Way ANOVA.

**Supplemental Figure S2. Pluripotency and differentiation marker expression in ciPSC and ciPSC-derived differentiated EBs.**

qRT-PCR showing downregulation of pluripotency markers and induction of differentiation markers upon differentiation of ciPSC. Normalization to undifferentiated ciPSC (ciPSC). EB: embryoid body differentiated ciPSC. n=2, Mean ± SD. * indicates significance with p<0.05, by Student t tests for each marker. Pluripotency markers: OCT4, SOX2, NANOG. Differentiation markers: KDR (mesoderm), AFP (endoderm), TUJ1 (ectoderm).

**Supplemental Figure S3. OCT4-eGFP reporter system construction.**

A) i) Cas9-sgRNA and ii) cOCT4-2A-eGFP-PGK-Puro donor plasmid constructs. iii) Schematic of the CRISPR/Cas9-mediated editing of the canine OCT4 locus. cHA: canine homology arm. B) i) Transfection efficiency shown as Relative mCherry Expression (count mCherry+ cells/DAPI nuclei) at Day 2 PTr; and ii) representative image of transfected CEF under Puromycin selection at Day 5 PTr, showing expression of mCherry (red). C) Sequencing results of the carboxyterminal end of the canine endogenous OCT4 locus, showing insertion of the GFP sequence and downstream editions. Representative sequencing of edition results on 2 different CEF lines (CEF-5 and CEF-3). PTr: post-transfection.

**Supplemental Figure S4. OKSIM-transduction efficiency in adult stromal cells CDF and cASC.**

Infection evaluated by OCT4 immunofluorescence at 48/72 hours post-transduction, defined as number of OCT4+ nuclei / number of DAPI+ nuclei. n = 10 for each group. Mean ± SEM.

**Supplemental Figure S5. Population Doubling Time for CDF, cASC and CEF cell types.**

Population doubling time expressed in hours. Mean ± SD. NS: Non-significant difference, as compared to CEF, p < 0.05, n=4.

**Supplemental Figure S6. Chromatin accessibility of pluripotency genes in stromal cells.**

Selected IGV genomic views of ATAC-seq data for stemness genes *SOX2*, *NANOG*, *OCT4*, *KLF4* and *MYC* for the three stromal cells CDF, cASC and CEF. All genome view vertical scales were group autoscaled to normalize for read-depth. Genes are oriented 5’-3’ and graphed from 2 kb upstream of the TSS to 2 kb downstream of the TES.

**Figure S7. Regulatory areas (promoter and 2kb upstream and downstream), exons and introns are enriched in peaks with the highest fold-change**

Proportion of peaks over total peaks, found in different genomic areas when considering either all peaks in the dataset or the highest FC peaks. FC: fold change.
