# Supplementary figures and images for: Chromatin accessibility in canine stromal cells and its implications for canine somatic cell reprogramming
Source: Stem Cells Transl Med. 2020 Nov 16;10(3):441–54. doi: 10.1002/sctm.20-0278 (PMC7900587; doi:10.1002/sctm.20-0278)

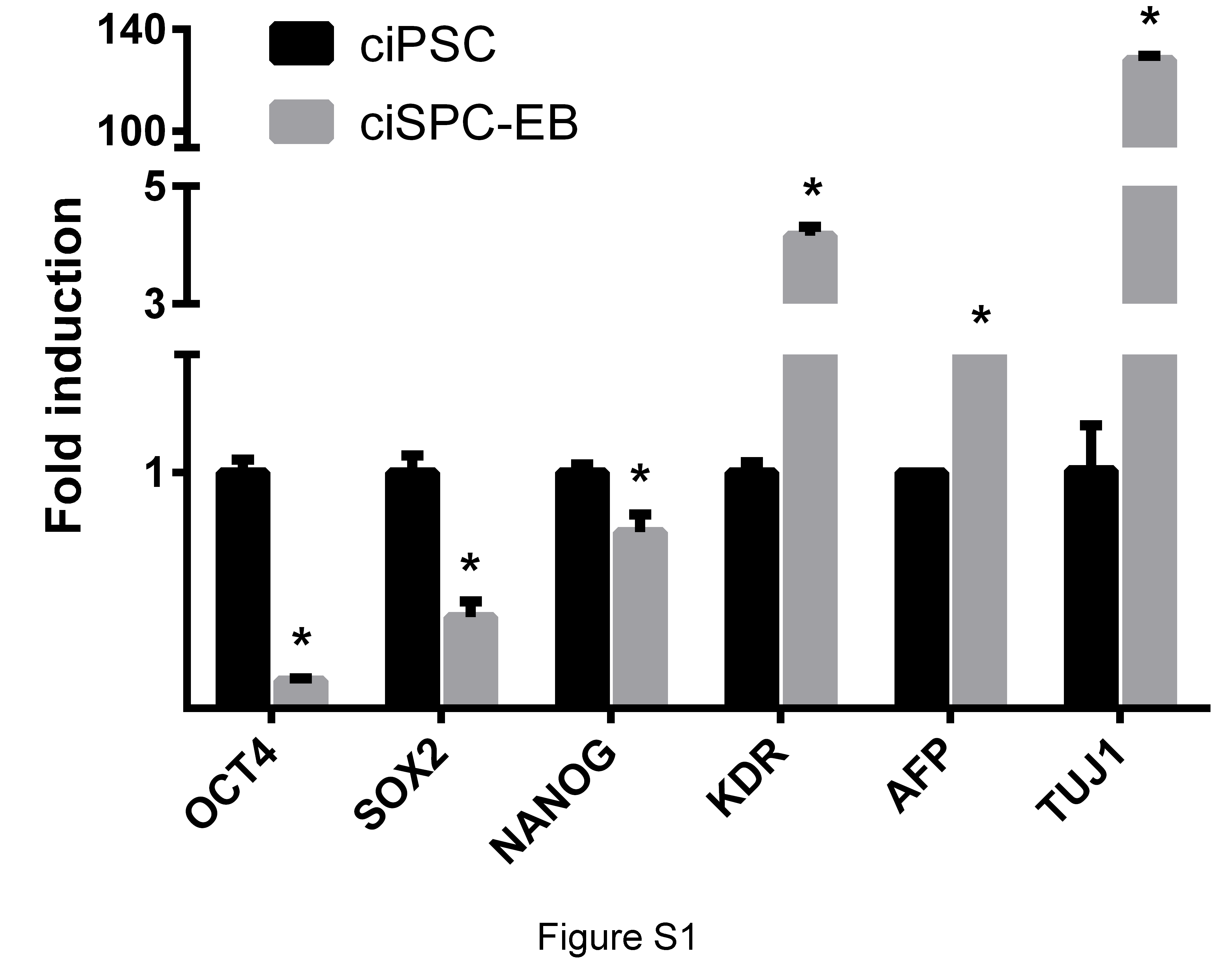

Supplement: Supplementary file 2 — FIGURE S1 Canine‐induced pluripotent stem cells shut down OKSM lentiviral transgene expression. qRT‐PCR showing repression of lentiviral transgenes in ciPSC after passage 15, when compared with a passage 4. Fold change expression of lentiviral cassette was assessed by primer pairs that bridge the OCT4 and KLF4, or KLF4 and SOX2 genes of the unique lentiviral transcriptional unit. Data represented is from n = 3 independent experiments, Mean ± SEM. Asterisk indicates significant difference with P < .01, when compared with ciPSC p4, by one‐way ANOVA. [file SCT3-10-441-s002.tif]

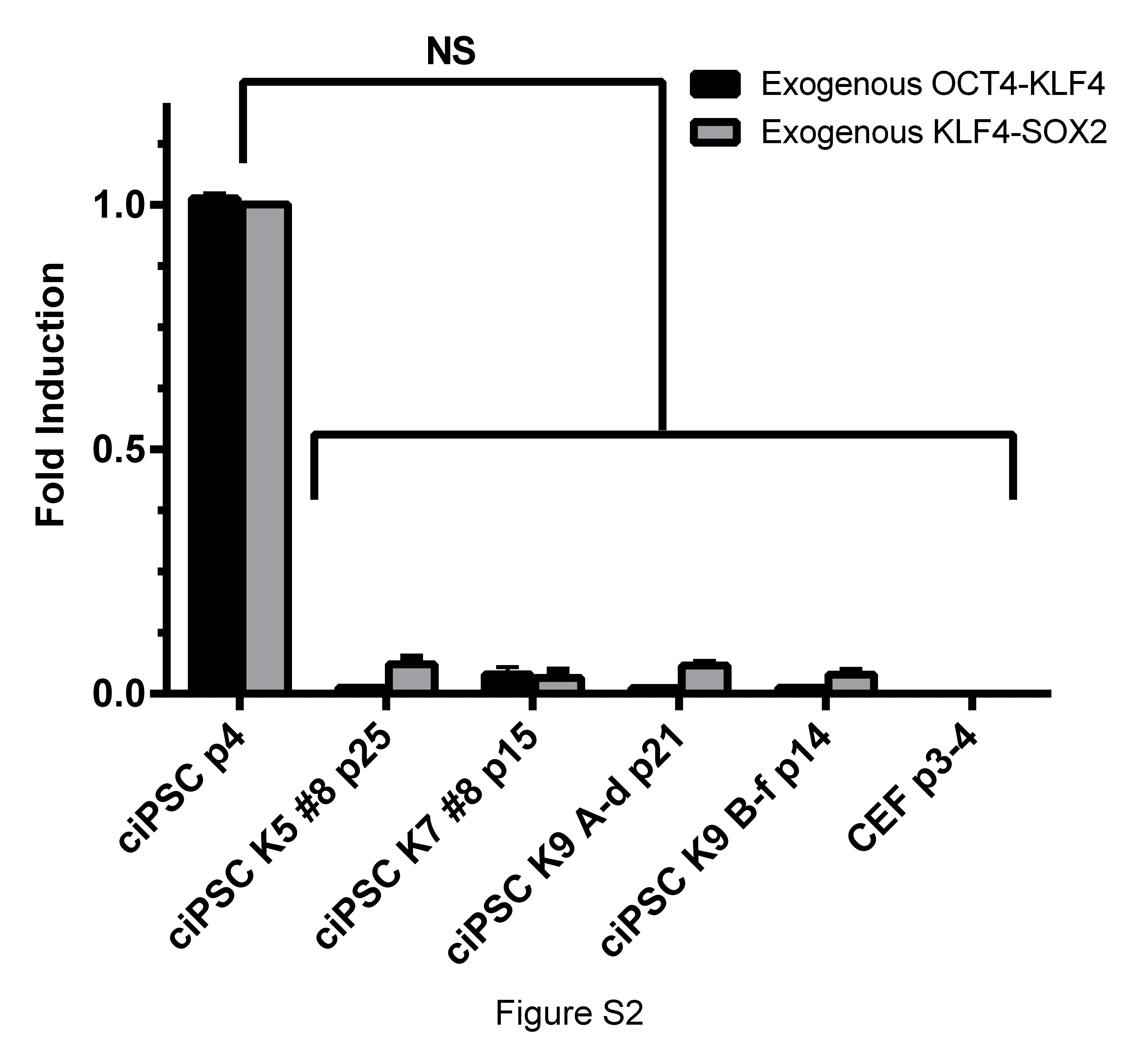

Supplement: Supplementary file 3 — FIGURE S2 Pluripotency and differentiation marker expression in ciPSC and ciPSC‐derived differentiated EBs. qRT‐PCR showing downregulation of pluripotency markers and induction of differentiation markers upon differentiation of ciPSC. Normalization to undifferentiated ciPSC (ciPSC). EB, embryoid body differentiated ciPSC. n = 2, Mean ± SD. Asterisk indicates significance with P < .05, by Student t tests for each marker. Pluripotency markers: OCT4, SOX2, NANOG. Differentiation markers: KDR (mesoderm), AFP (endoderm), TUJ1 (ectoderm). [file SCT3-10-441-s003.tif]

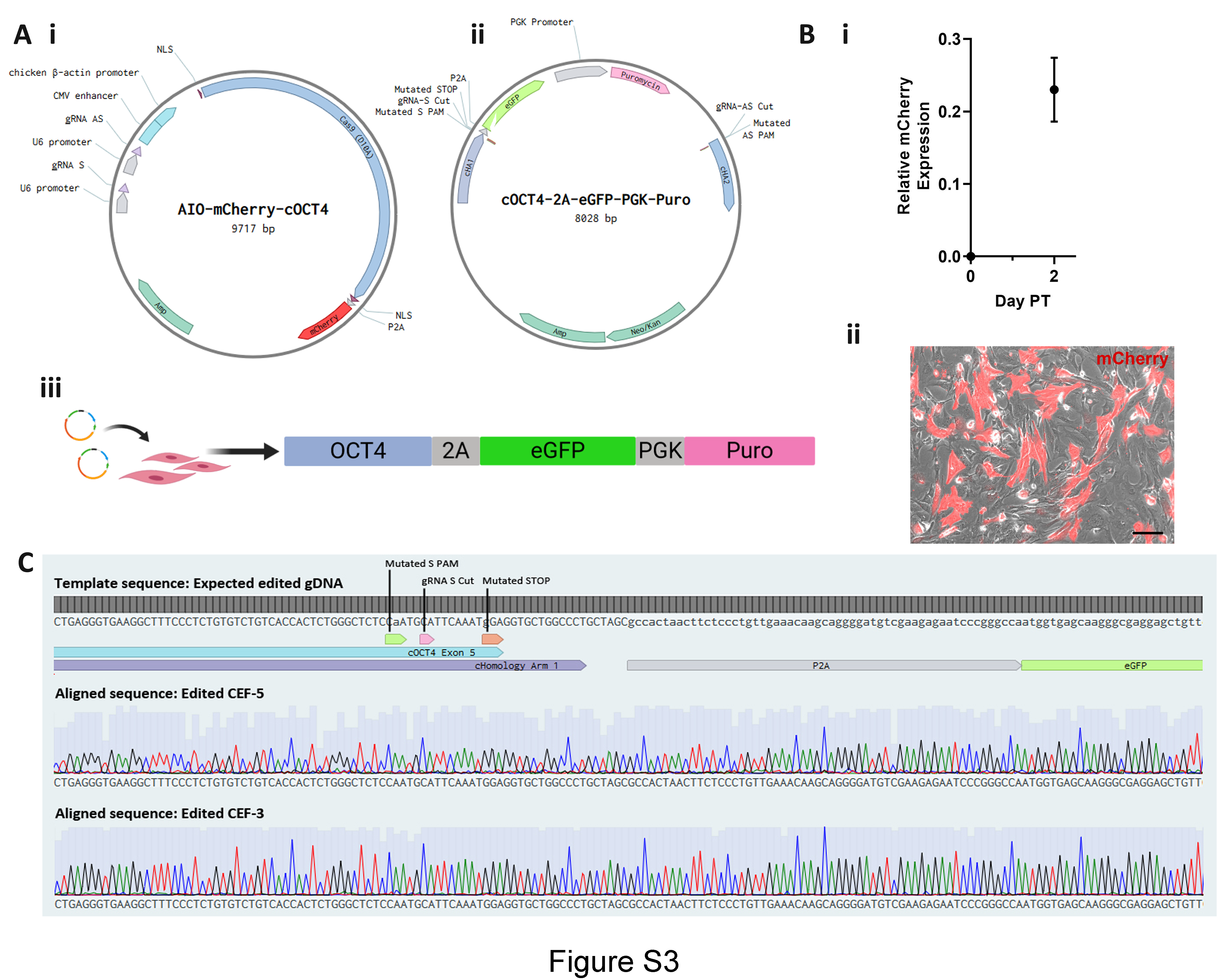

Supplement: Supplementary file 4 — FIGURE S3 OCT4‐eGFP reporter system construction. A, (i) Cas9‐sgRNA and (ii) cOCT4‐2A‐eGFP‐PGK‐Puro donor plasmid constructs. (iii) Schematic of the CRISPR/Cas9‐mediated editing of the canine OCT4 locus. cHA, canine homology arm. B, (i) Transfection efficiency shown as Relative mCherry Expression (count mCherry+ cells/DAPI nuclei) at day 2 PTr; and (ii) representative image of transfected CEF under puromycin selection at Day 5 PTr, showing expression of mCherry (red). C, Sequencing results of the carboxyterminal end of the canine endogenous OCT4 locus, showing insertion of the GFP sequence and downstream editions. Representative sequencing of edition results on two different CEF lines (CEF‐5 and CEF‐3). PTr, posttransfection. [file SCT3-10-441-s004.tif]

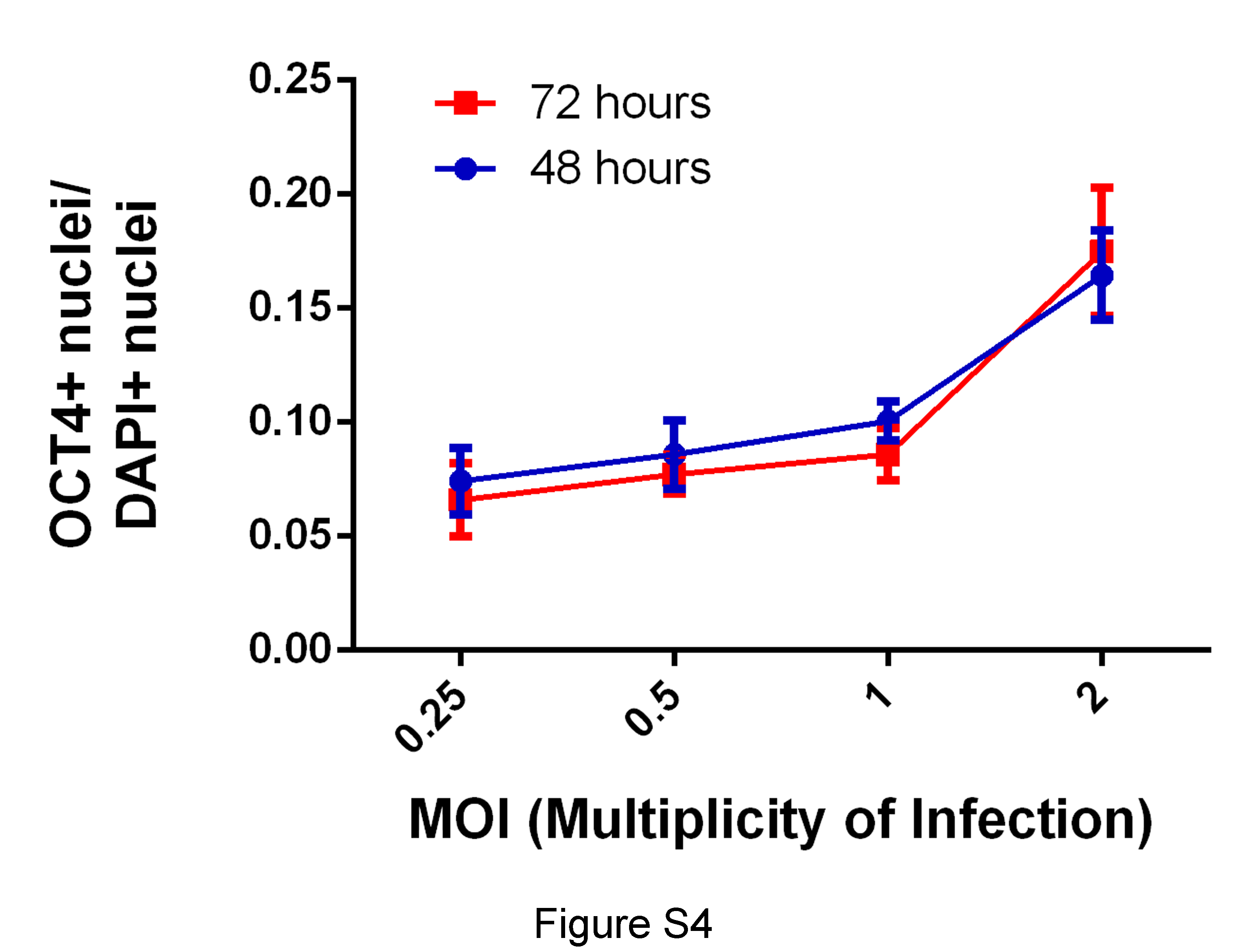

Supplement: Supplementary file 5 — FIGURE S4 OKSIM transduction efficiency in adult stromal cells CDF and cASC. Infection evaluated by OCT4 immunofluorescence at 48/72 hours post‐transduction, defined as number of OCT4+ nuclei/number of DAPI+ nuclei. n = 10 for each group. Mean ± SEM. [file SCT3-10-441-s005.tif]

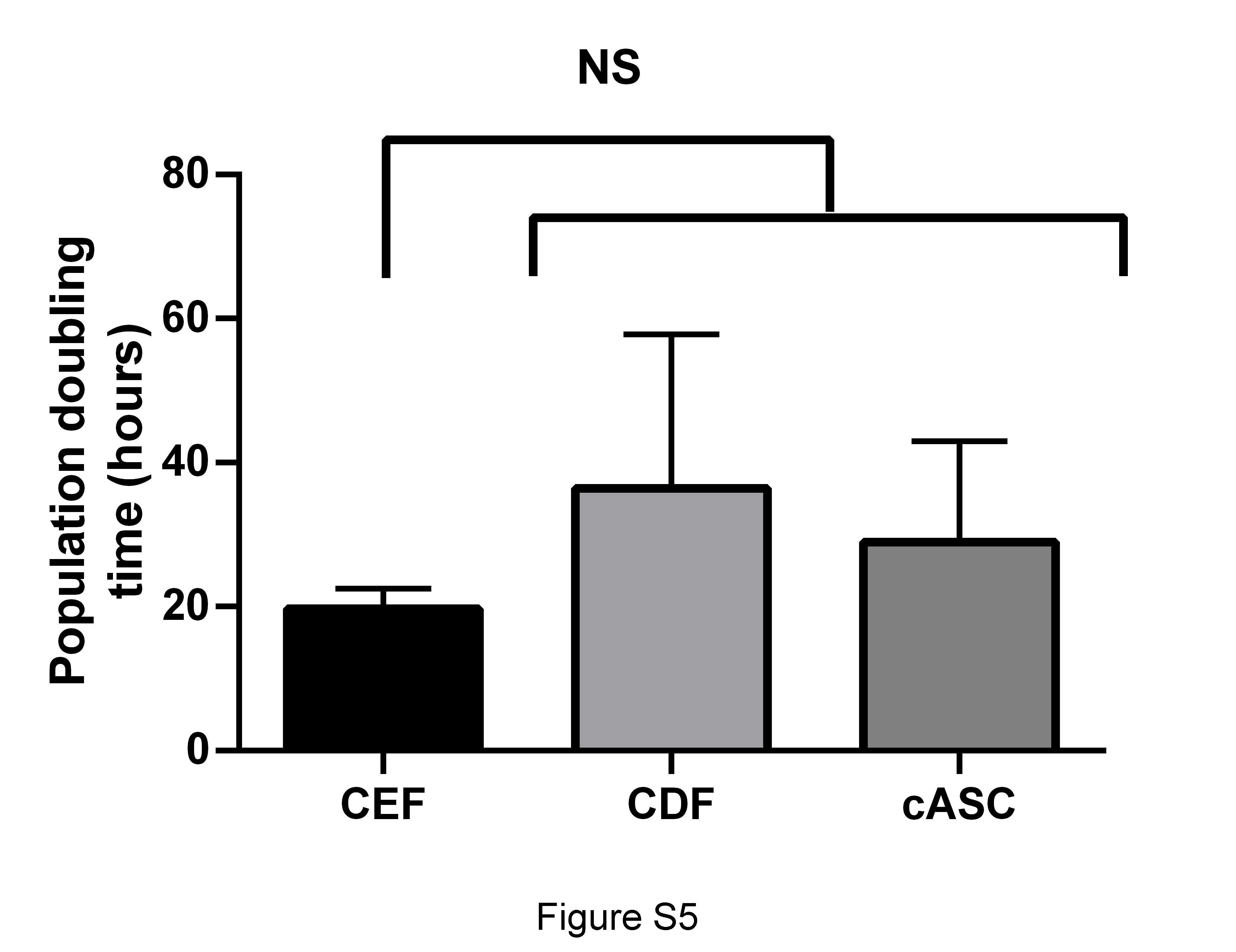

Supplement: Supplementary file 6 — FIGURE S5 Population Doubling Time for CDF, cASC and CEF cell types. Population doubling time expressed in hours. Mean ± SD. NS, nonsignificant difference, as compared with CEF, P < .05, n = 4. [file SCT3-10-441-s006.tif]

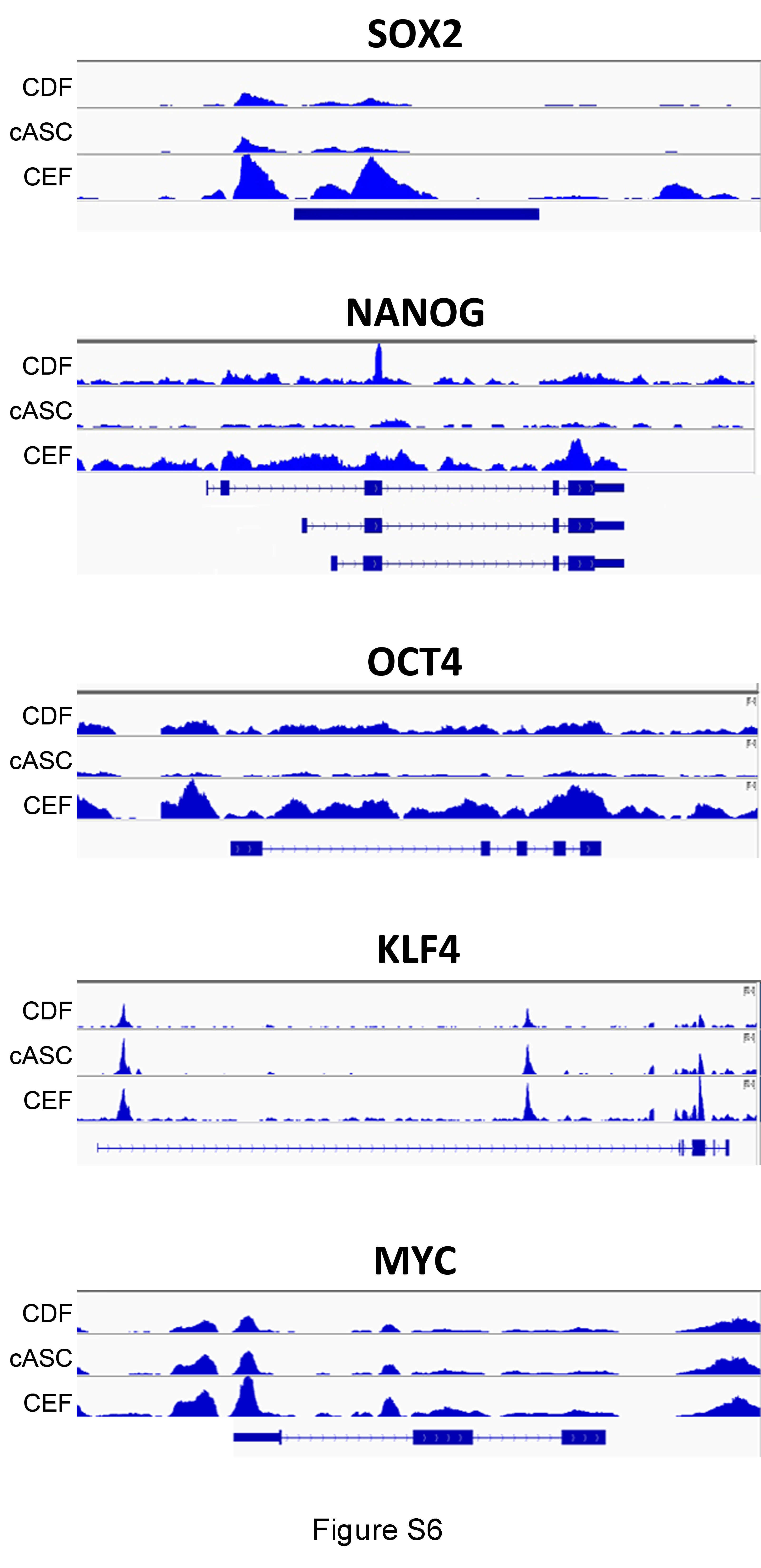

Supplement: Supplementary file 7 — FIGURE S6 Chromatin accessibility of pluripotency genes in stromal cells. Selected IGV genomic views of ATAC‐seq data for stemness genes SOX2, NANOG, OCT4, KLF4, and MYC for the three stromal cells CDF, cASC and CEF. All genome view vertical scales were group autoscaled to normalize for read‐depth. Genes are oriented 5′‐3′ and graphed from 2 kb upstream of the TSS to 2 kb downstream of the TES. [file SCT3-10-441-s007.tif]

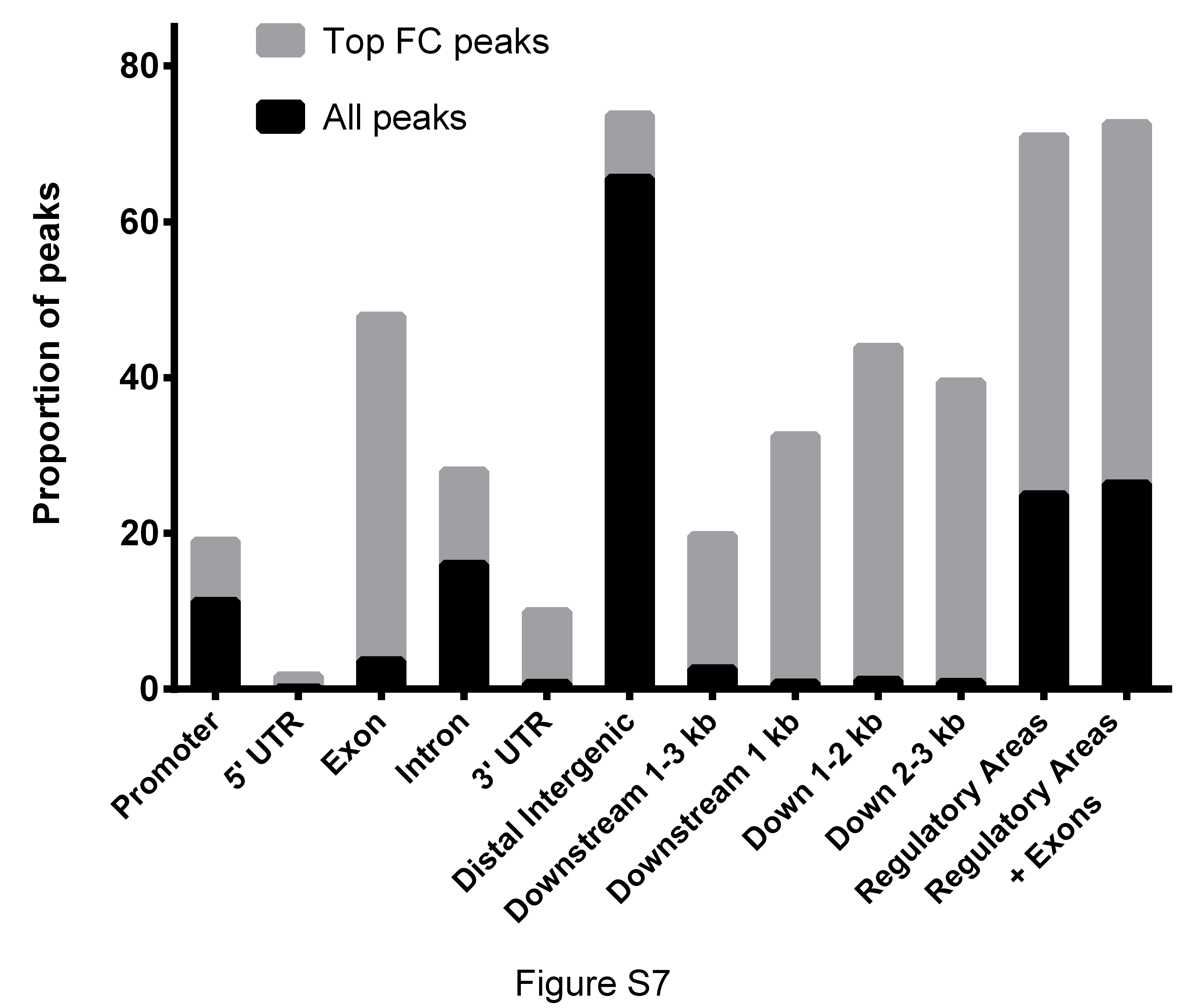

Supplement: Supplementary file 8 — FIGURE S7 Regulatory areas (promoter and 2 kb upstream and downstream), exons and introns are enriched in peaks with the highest fold‐change. Proportion of peaks over total peaks, found in different genomic areas when considering either all peaks in the data set or the highest FC peaks. FC, fold change. [file SCT3-10-441-s008.tif]
