# Supplementary material for: Chromatin accessibility in canine stromal cells and its implications for canine somatic cell reprogramming
Source: Stem Cells Transl Med. 2020 Nov 16;10(3):441–54. doi: 10.1002/sctm.20-0278 (PMC7900587; doi:10.1002/sctm.20-0278)
Supplement: Supplementary file 9 — TABLE S1 Breed, age and sex of canine individuals per cell line. TABLE S2. List of primers used for qRT‐PCR. TABLE S3. List of antibodies used for immunofluorescence. [file SCT3-10-441-s009.docx]

**Supplemental Tables**

| **Cell Line** | **Breed** | **Age (CDF/cASC)**  **Fetal Age (CEF)** | **Sex** |
| --- | --- | --- | --- |
| CDF-1 | Irish Terrier | 8 years old | Male |
| CDF-2 | Yorkshire Terrier | 11 years old | Female |
| CDF-3 | Chihuahua | 12 years old | Female |
| CDF-6 | Whippet | 8 years old | Male |
| cASC Cheela | Chihuahua | 10 year old | Female |
| cASC DJ | Beagle | 10 year old | Male |
| cASC Milo | Lhasa/Chihuahua cross | 9 month old |  |
| cASC Topaz | Crossbreed | 2 years old | Male |
| CEF-3 | Crossbreed | 30-50 days gestation | Female |
| CEF-4 | Crossbreed | 30-50 days gestation | Male |
| CEF-8 | Crossbreed | 21-28 days gestation | Female |
| CEF-9 | Crossbreed | 21-28 days gestation | Male |

**Table S1. Breed, age and sex of canine individuals per cell line.**

| **Gene/Sequence** | **Amplicon size (bp)** | **Primer Sequences** |
| --- | --- | --- |
| *GAPDH* | 100 | F: CATGTTTGTGATGGGCGTGAACCA  R: TTTGGCTAGAGGAGCCAAGCAGTT |
| *NANOG* | 138 | F: ACAGAAGATGAGGACGGTGTTC  R: GTTTTAACCTGCTTATAGCTGAGGT |
| *OCT4* | 166 | F: CGAGGAGTCCCAAGACATCAAA  R: CTCAAAACGGCAGATGGTTGTT |
| *SOX2* | 143 | F: GTCCCAGCACTACCAGAGCG  R: CTTACTCTCCTCCCATTTCCCTCG |
| *OK* | 158 | F: TCTCCCATGCATTCAAACG  R: GTGGAGAAAGATGGGAGCAG |
| *KS* | 119 | F: TCGGACCACCTCGCCTTACACATGA  R: GGATTGCTTTCTACATCCCCAGCCA |

**Table S2. List of primers used for qRT-PCR.**

| **Antigen target** | **Manufacturer** | **Catalogue No.** | **Type** | **Concentration** |
| --- | --- | --- | --- | --- |
| AFP | Dako | A 0008 | Polyclonal | 1:2000 |
| GFAP | Cell Signaling Technology | 12389 | Monoclonal | 1:200 |
| KDR | Millipore | 07-716-I | Polyclonal | 1:400 |
| NANOG | Abcam | Ab77095 | Polyclonal | 1:100 |
| OCT4 | Santa Cruz Biotechnology | SC-5279 | Monoclonal | 1:200 |
| SOX2 | Millipore | AB5603 | Polyclonal | 1:100 |
| TUJ1 | BioLegend | 801201 | Monoclonal | 1:300 |
| VIM | Dako | M7020 | Monoclonal | 1:300 |

**Table S3. List of antibodies used for immunofluorescence.**
